# DSS1 interacts with and stimulates RAD52 to promote the repair of DSBs

**DOI:** 10.1093/nar/gkz1052

**Published:** 2019-12-04

**Authors:** Barbora Stefanovie, Sarah R Hengel, Jarmila Mlcouskova, Jana Prochazkova, Mario Spirek, Fedor Nikulenkov, Daniel Nemecek, Brandon G Koch, Fletcher E Bain, Liping Yu, Maria Spies, Lumir Krejci

**Affiliations:** 1 Department of Biology, Masaryk University, 62500 Brno, Czech Republic; 2 International Clinical Research Center, St. Anne's University Hospital in Brno, 62500 Brno, Czech Republic; 3 Department of Biochemistry, University of Iowa Carver College of Medicine, 51 Newton Road, Iowa City, IA 52242, USA; 4 CEITEC, Masaryk University, 62500 Brno, Czech Republic; 5 NMR Core Facility, Carver College of Medicine, University of Iowa, Iowa City, IA 52242, USA; 6 National Centre for Biomolecular Research, Masaryk University, 62500 Brno, Czech Republic

## Abstract

The proper repair of deleterious DNA lesions such as double strand breaks prevents genomic instability and carcinogenesis. In yeast, the Rad52 protein mediates DSB repair via homologous recombination. In mammalian cells, despite the presence of the RAD52 protein, the tumour suppressor protein BRCA2 acts as the predominant mediator during homologous recombination. For decades, it has been believed that the RAD52 protein played only a back-up role in the repair of DSBs performing an error-prone single strand annealing (SSA). Recent studies have identified several new functions of the RAD52 protein and have drawn attention to its important role in genome maintenance. Here, we show that RAD52 activities are enhanced by interacting with a small and highly acidic protein called DSS1. Binding of DSS1 to RAD52 changes the RAD52 oligomeric conformation, modulates its DNA binding properties, stimulates SSA activity and promotes strand invasion. Our work introduces for the first time RAD52 as another interacting partner of DSS1 and shows that both proteins are important players in the SSA and BIR pathways of DSB repair.

## INTRODUCTION

To avoid genome instability, a hallmark and enabling characteristic of cancer (1), cells need to carry out efficient replication and repair when DNA lesions such as double-stranded breaks (DSBs) occur. Many critical players are shared during cellular mechanisms that promote DNA replication completion, mediate replication fork recovery and restart damaged replication forks, and repair DSBs via homologous recombination (HR) (2–5).

In yeast, HR primarily depends on proteins within the *RAD52* epistasis group (6). Among all members of this epistasis group deletion of the *RAD52* gene in *S. cerevisiae* leads to the strongest HR and DNA repair phenotype, accentuating its importance. The yeast Rad52 protein is a recombination mediator as it facilitates nucleation of the Rad51 filaments on ssDNA bound by the ssDNA binding protein RPA (7,8). In mammalian cells, the BRCA2 tumour suppressor protein plays a central HR function by mediating formation of RAD51 presynaptic filament required for DSB repair (9,10) and protection of stalled replication forks (11,12).

The human RAD52 protein plays an important yet historically elusive role in DNA repair. Initial characterization identified roles in SSA and second-end capture during RAD51-dependent DSB repair (13,14). Depletion or pharmacological inhibition of human RAD52 has a synthetically lethal relationship with defects in both BRCA2 (15–19) and BRCA1/PALB2 (20). This relationship, however, cannot be fully explained by HR defects alone, as RAD52 does not compensate for BRCA2 deficiency with respect to HR. Moreover, depletion of RAD52 only has a mild effect on HR (21,22). Instead of functioning in HR, RAD52 in mammalian cells is required for the repair (23) and restart (24) of stalled replication forks, for mitotic DNA synthesis (MIDAS) (25), SSA (38) and BIR events (24,26). Additionally, RAD52 plays a gatekeeper function at stalled replication forks where it antagonizes fork reversal by SMARCAL1 (27). Furthermore, RAD52 has been found to be important for repair of >50 nt repeat sequences that flank DSBs and combined depletion with POLQ cause hypersensitivity to cisplatin and a synthetic reduction in replication fork restart (28). Structurally, the human RAD52 protein forms oligomers with an average of seven oligomers (29,30). The RAD52 monomer consists of two domains, an evolutionarily conserved N-terminal domain (NTD) and species specific C-terminal domain (CTD) (31). The NTD is involved in DNA binding and contains an oligomerization domain (32,33), while the CTD harbors RPA and RAD51 interaction domains (34,35). The RAD52 protein harbors two DNA binding sites. The inner DNA binding site binds ssDNA within a positively charged groove spanning the circumference of the ring (33,36) and the outer DNA binding site lies above the inner DNA binding site and binds both ssDNA and dsDNA (37). This unique binding mode may facilitate single-strand annealing of complementary ssDNA (38).

The BRCA2 protein functions in complex with the highly conserved, small, and very acidic protein DSS1 to promote the RAD51-loading activity of BRCA2 (39). Moreover, the binding of DSS1 masks a nuclear export signal of BRCA2 and thereby controls both BRCA2 and RAD51 nuclear localization (40). Recently, DSS1 was also shown to promote BRCA2-dependent HR by targeting RPA. It was suggested that DSS1 could mimic DNA and reduce the affinity of RPA for ssDNA, thereby facilitating a handoff of ssDNA from RPA to RAD51 (41). Despite the newly identified DSS1 interaction proteins within HR pathway, how DSS1 cooperates with multiple genome maintenance proteins in many diverse processes remains unknown. Similarly, the functional relationship between BRCA2 and RAD52 remains unclear.

Here, we show that the RAD52 protein is a novel interacting partner of DSS1. This interaction changes the RAD52 protein conformation and modulates DNA binding resulting in stimulated annealing and D-loop activities of RAD52. We show that DSS1 acts not only in the BRCA2-mediated HR pathway, but also in RAD52-dependent SSA and BIR repair pathways. We propose that DSS1 and RAD52 function together in SSA but seem to have separate roles in BIR.

## MATERIALS AND METHODS

### Protein purifications

The pGEX-KG plasmid carrying GST-DSS1 (Supplementary Table S1) was introduced into *Escherichia coli* BL21 (DE3) cells (New England BioLabs). Cells were grown at 37°C until OD_600_ = 0.6 and the GST-DSS1 expression was induced by addition of 0.5 mM IPTG and incubation at 37°C for 4 h. Harvested cells were resuspended in T+300 buffer (25 mM Tris–HCl pH 7.5, 300 mM KCl, 10% glycerol, 0.5 mM EDTA, 0.01% NP40, 1 mM DTT, cocktail of protein inhibitors), followed by sonication in order to disrupt the DNA and finally centrifuged for 1 h at 35 000 × g at 4°C. Supernatant was mixed with 1 ml of Glutathione 4B-Sepharose beads (GE Healthcare) equilibrated in T+300 buffer for 1 h at 4°C. A gravity column was used to remove unbound fraction and beads were washed with 10 ml of T+300 buffer to remove non-specifically bound proteins. Bound protein was eluted by 2 × 1 ml of T+300 buffer containing 10, 50 or 100 mM glutathione respectively. Both 50 mM glutathione fractions were pooled, diluted with T buffer to decrease the conductivity and loaded onto 1 ml-MonoQ (GE Healthcare) equilibrated in buffer T supplemented with 100 mM KCl. GST-DSS1 was eluted with 10 column volumes gradient of the 100–1000 mM KCl in buffer T containing 1000 mM KCl. Fractions from 440 to 480 mM KCl were pooled, split into halves and first half was concentrated on VivaSpin 2.0 (MWCO 5000) to 150 μl and washed two times in T+100 buffer. GST tag in the second half of protein was cleaved by 30 units of thrombin (Sigma-Aldrich) O/N at RT. Cleavage was stopped by the addition of 1 mM PMSF and the mixture of cleaved GST and DSS1 was diluted in T buffer and loaded onto 0.5-ml Mono Q equilibrated in buffer T containing 100 mM KCl. DSS1 was then eluted by 20 column volume gradient of 200–700 mM KCl. Peak fractions were pooled, concentrated on VivaSpin 2,0 (MWCO 5000) and washed in T+100 buffer to decrease salt concentration. Both proteins were aliquoted and stored at −80°C.

The RAD52 protein was overexpressed using pET11a vector (Supplementary Table S1) in *E. coli* Rosetta™ (DE3) pLysS cells (Novagen). Protein expression was induced by 0.5 mM IPTG and incubation at 16°C overnight. Harvested cells were resuspended in K buffer (20 mM KH_2_PO_4_, 500 mM KCl, 10% glycerol, 0.5 mM EDTA, 0.01% NP40, 1 mM β-mercaptoethanol) containing 500 mM KCl, followed by sonication and centrifugation for 1 h at 35 000 × g at 4°C. Supernatant was diluted with K buffer and loaded on 30-ml SP Sepharose column (GE Healthcare) equilibrated in K+150 buffer. RAD52 was eluted by 240 ml of 3–65% KCl gradient of buffer K+1000. Fractions containing RAD52 protein were pooled, diluted and loaded on 1-ml Heparin Fast Flow (GE Healthcare) followed by 10 column wash and elution with 10 ml of 5–75% KCl gradient of K+1000 buffer. Fractions form 360–430 mM KCl containing the majority of RAD52 protein was pooled, diluted with K buffer and loaded on 0.5-ml Mono S column. RAD52 protein was eluted with 10 ml gradient of 5–55% of K+1000. Fractions containing protein (in the range of 300–350 mM KCl) were pooled and concentrated on VivaSpin 2.0 (MWCO 30 000). Size exclusion chromatography was further used to increase the homogeneity of the protein sample. Sample was loaded on 25-ml Sephacryl S-400 (GE Healthcare) equilibrated in K+150 buffer and eluted with 25 ml of K+150 buffer. Fraction containing RAD52 were pooled, concentrated, aliquoted and stored at −80°C.

The His6-RAD52 and yeast His-Rad52 proteins were expressed and purified as described before (42,43). There are extensive evidences that the N-terminal tag, including His-tag, does not affect the activity (44).

### Recombinant Pull-down assays

Glutathione Sepharose™ 4B resin (20 μL, GE Healthcare) was pre-equilibrated in T+100 buffer. Reaction mixture containing GST-DSS1 (5 μg) and RAD52 (5 μg) proteins in 30 μl of T+100 buffer was added to the resin and incubated for 30 min in the Thermomixer (Eppendorf) at 1150 × rpm at 4°C. Samples were centrifuged and flow through fraction (FT) collected. Resin was washed two times with 200 μl of T+100 buffer. The bound fraction(B) was eluted by addition of 20 μl of SDS-Laemmli buffer and all samples were analysed by 12% SDS-PAGE.

In the quantitative GST-DSS1 pulldown experiments, GST-DSS1 (at a final concentration of 4 μM) was mixed with the indicated concentrations of His6-RAD52 in the binding buffer (20 mM Tris-acetate pH 7.5, 100 mM KCl, 0.01% NP40, 1 mM DTT) and GST beads (Pierce & Thermo Scientific) in binding buffer (20 mM Tris-acetate pH 7.5, 100 mM KCl, 0.01% NP40, 1 mM DTT) to a final volume of 30 μl. Beads and proteins were incubated for 1 h rotating at 4°C. Beads and proteins were pelleted at 3,000 × g for 5 minutes, washed twice with 200 μl of binding buffer. Bound proteins were eluted by adding 5 μl of binding buffer and 5 μl of Laemmli buffer and boiling for ∼10 min. Proteins were separated using a 12% SDS-Page gel (200 V, 50 min). Quantification was carried out using volume intensity analysis using BIORAD Image Software and the data was normalized to the 6 μM His6-RAD52 intensity value. All data was plotted using GraphPad Prism 7.

### NMR Spectroscopy

NMR spectra were acquired on a 500 or 800 MHz Bruker Avance II NMR spectrometer at 5°C using a 220 μM uniformly [^15^N,^13^C]-labelled DSS1 for backbone and sidechain assignments or a 10 μM uniformly ^15^N-labelled DSS1 for binding experiments with RAD52 in a buffer containing 25 mM Tris, 50 mM KCl and 25 μM EDTA, pH 7.5 in 90% H_2_O/10% D_2_O. A suite of triple resonance NMR experiments including HNCACB, HN(CO)CACB, HNCO, HN(CA)CO, HNCA and HN(CO)CA experiments (45) were acquired for backbone assignments of DSS1. Sidechain assignments were obtained by acquiring C(CO)NH, H(CCO)NH, HBHA(CO)NH and ^15^N-NOESY spectra (46). ^15^N-heteronuclear NOEs and ^15^N T_2_ relaxation times of DSS1 were obtained as described previously (47). Duplicates of ^15^N{^1^H}-heteronuclear NOE experiments were performed and used to calculate the average value and standard deviation for each backbone amide. The standard deviations of ^15^N T_2_ relaxation times of DSS1 were obtained from data fitting. The RAD52 binding experiments were performed by acquiring a series of ^15^N/^1^H HSQC spectra of 10 μM uniformly ^15^N-labelled DSS1 in the absence of His6-RAD52 and in the presence of 2.5, 5 and 10 μM His6-RAD52. The acquired data were analysed by peak heights. The ^1^H and ^13^C chemical shifts were referenced to 2,2-dimethyl-2-silapentane-5-sulfonate. The collected data were processed using NMRPipe (48) and analysed using NMR View (49).

### Microscale thermophoresis

For the purpose of MST measurements, DSS1 was labelled by NT-647-NHS dye according to the manufacturer protocol (NanoTemper Technologies). Constant concentration of labelled DSS1 protein (50 nM) was mixed with increasing concentrations of RAD52 (1 nM to 36 μM) in Tris-Acetate buffer (30 mM Tris-acetate pH 7.5, 1 mM DTT) in a series of 16 independent capillaries. Mixed proteins were incubated at 25°C for 10 min and measured using low MST power and 20% of Excitation power. Changes in fluorescence were plotted against RAD52 concentration and *K*_d_ value was calculated using quadratic binding equation for six independent measurements at two different MST power.

### Electromobility shift assay (EMSA)

Increasing concentrations of RAD52 protein or the RAD52-DSS1 complex (pre-formed in 1:1 ratio by incubation in Tris-acetate buffer (30 mM Tris-acetate pH 7.5, 1 mM DTT) for 10 min at 4°C were mixed with 15 nM fluorescently labelled DNA substrate (pR1027, Supplementary Table S2) in the reaction buffer. Reactions were incubated for 10 min at RT and subsequently cross-linked by 0.1% glutaraldehyde for 10 min at RT. Reaction products were resolved on 0.8% 1× TAE agarose gel in TAE buffer, visualized by Image Reader FLA-9000 and quantified using MultiGauge V3.2 software (Fujifilm). Reported values are averages of three independent experiments.

### FRET-based ssDNA and phiX binding assays

FRET-based DNA binding assays were carried out as previously described (50) using a Cary Eclipse spectrophotometer (Varian) at 25°C. Briefly, the FRET-based DNA binding assays were performed in reaction buffer (30 mM Tris-acetate, 1mM DTT, pH 7.5 and 0.1 mg/ml BSA) in a 5 mm cuvette with a reaction volume of 600 μl. After measuring the baseline, 1 nM Cy3-dT_30_-Cy5 oligonucleotide (Supplementary Table S2) was added into the cuvette followed by addition of the indicated concentrations of His6-RAD52. After each titrant the solution was mixed and allowed to reach equilibrium. The equilibrium fluorescence was recorded for 2 min in the Cy3 and Cy5 channels. The data were averaged after each addition and background was subtracted. For each of the protein concentrations apparent FRET was calculated as described previously (50). Each assay was performed in triplicate and the average of each FRET value was then plotted against the concentration of RAD52 (monomers). For titrations with pre-mixed His6–RAD52–DSS1 complexes, the two proteins were mixed at 1:20 ratio and incubated at 4°C for 5 min. For competition assays with dsDNA, the indicated amount of dsDNA (ΦX174 RF I DNA, Supplementary Table S1) digested with ApaLI, was titrated into the cuvette containing stoichiometric His6-RAD52/Cy3-dT_30_-Cy5 or His6-RAD52/DSS1/Cy3-dT_30_-Cy5 complexes. Reported values are averages of at least three independent experiments.

### Single-strand annealing assay

The 3′-overhang substrates with 32 nucleotides complementary ssDNA were prepared by annealing in Hybridization buffer (50 mM HEPES, 150 mM NaCl, pH 7.5). Labelled oligonucleotide pR101 (Supplementary Table S2) was mixed with the 1.5× excess of unlabelled oligonucleotide pR2863 (Supplementary Table S2) and the reaction was heated to 95°C for 5 min and allowed slowly cool to reach RT. The completion of the self-annealing reactions was checked on 13% native PAGE in 1xTBE. Reaction mixtures containing increasing concentrations of either RAD52 or pre-formed RAD52-DSS1 complex (1:1) were incubated with 3 nM fluorescently labelled 3′-overhang substrates (pR101 + pR2863) for 3 min at room temperature. Complementary 3′-overhang substrate (6 nM, pR2864 + pR2865, Supplementary Table S2) was then added to reactions and incubated for another 10 min at RT. The reactions were deproteinized using 0.1% SDS and 500 μg/ml Proteinase K (PanreacAppliChem) for 10 min at RT. Products were resolved on 10% native PAGE in 1× TBE, visualized by Image Reader FLA-9000 and quantified using MultiGauge V3.2 software (Fujifilm). Reported values are averages of three independent experiments.

### FRET-based annealing assays

FRET-based annealing of complementary oligonucleotides by RAD52 was monitored under identical conditions as the FRET binding assays described above. For each assay, the reaction master mixture containing RAD52 protein (8 nM) in the presence and absence of DSS1 protein (40 nM) were prepared at room temperature and divided into two half reactions. Following baseline buffer and protein measurements, 0.5 nM of Target-28Cy3 ssDNA substrate (Supplementary Table S2) was added to the reaction cuvette and the signal was allowed to stabilize. The annealing reaction was initiated upon addition and mixing of the second half-reaction pre-incubated with 0.5 nM Probe-28Cy5 (Supplementary Table S2) ssDNA substrate. Reactions with RPA were performed by pre-incubating 2 nM RPA with 0.5 nM ssDNA substrate (saturating amount of RPA was used, 1 molecule of RPA per 28 nts ssDNA) before addition of RAD52 or the RAD52-DSS1 complex. The fluorescence of Cy3 and Cy5 were measured simultaneously over the reaction time course (150s). Apparent FRET values were calculated as an average of three or more independent annealing reactions plotted against time (s). The average FRET values were fitted to a double exponential equation.

### D-loop assay

Increasing concentration of RAD52 or RAD52–DSS1 complex (1:1) (150, 300, 600, 800, 1600 and 3200 nM) was pre-incubated with fluorescently labelled oligonucleotide (pR231, 30 nM, Supplementary Table S2, Mw = 28.295 kDa, 8.46 ng in reaction) in Borate buffer (20 mM Borate, 75 mM KCl, pH 7.5) for 3 min at 37°C. Subsequently, the formation of the D-loop product was initiated by addition of 540 ng pBluescript II SK plasmid DNA (Supplementary Table S1, 2 μl of 270 ng/μl in 10 μl of total reaction volume) and incubated for 5 min at 37°C. The molar ration between oligonucleotide and duplex target was ∼1:1. For the RAD51 protein control (in the concentration of 1 and 2 μM), reaction mixture contained extra 1 mM ATP and 2 mM CaCl_2_. Reactions were deproteinized by incubation with 0.1% SDS and 500 μg/ml of Proteinase K (PanreacAppliChem) for 5 min at RT. Products were resolved on 0.8% 1× TAE agarose gel, visualized by Image Reader FLA-9000 and quantified using MultiGauge V3.2 software (Fujifilm). Reported values are averages of three independent experiments.

### S1 nuclease protection assay

Fluorescently labelled oligonucleotide (pR1027, 15 nM, Supplementary Table S2) was pre-incubated with 960 nM RAD52 or pre-formed stoichiometric RAD52-DSS1 complex (1:1) in Tris-acetate buffer (30 mM Tris-Acetate, 1 mM DTT, pH 7.5) for 10 min at RT. Subsequently, 0.2 U of S1 nuclease (Takara) was added to the reaction and aliquots were withdraw at 5, 15 and 60 min. Reactions were deproteinized by incubation with 0.1% SDS and 500 μg/ml of Proteinase K (Panreac AppliChem) for 10 min at RT. Products were resolved on 30% denaturing PAGE gel, visualized by Image Reader FLA-9000 and quantified using MultiGauge V3.2 software (Fujifilm). Reported values are averages of three independent experiments.

### Cell culture

The human U2OS osteosarcoma cell lines (Supplementary Table S4) were grown in Dulbecco's modified Eagle's medium (DMEM, high glucose, Glutamax) containing 10% FBS and penicillin-streptomycin antibiotics under standard cell culture conditions (5% CO_2_, humidified atmosphere).

### Cell-based GFP-reporter assays

U2OS cell lines with a stable integrated reporter constructs for monitoring either DR, SSA (Supplementary Table S4) were obtained as a kind gift from Dr.Jeremy Stark and were described elsewhere (51). GFP-reporter U2OS cell line monitoring BIR repair (Supplementary Table S4) was received from Dr Thanos Halazonetis and its construction described previously (52). Cells were seeded to a density of 3 × 10^5^ per well in six-well plates and reverse transfected with siRNAs (Supplementary Table S3). Next day, cells were split to 12-well plates in triplicates and following day transfected with 0.3 μg of I-SceI plasmid DNA (Supplementary Table S1) using PEI in 6:1 ratio. Expression of GFP reporter was monitored by flow cytometry 48 h post transfection. Reported values are averages of three independent experiments.

### Nuclei staining, cell fixation and fluorescence microscopy

For ionizing radiation (IR) induced U2OS-YFP-RAD52 foci, cells were grown on IBIDI 35 mm μ-dishes and incubated overnight. Next day, cells were irradiated (10 Gy) and 2 h post irradiation fixed with 4% formaldehyde at room temperature for 10 min, washed three times with PBS and nuclei were stained using DAPI (Panreac AppliChem). For hydroxyurea (HU) induced U2OS-YFP-RAD52 foci, cells were grown on IBIDI eight-well μ-slides and incubated overnight. Next day, cells were treated with 2 mM HU (sigma-Aldrich) for 4 h and fixed as described above. The slides were viewed at 600× magnification on Nikon fluorescence microscope (EclipseTi-E) using high-throughput mode with 100 images captured for one sample. Nd files from Nikon Eclipse Ti-E was used in automated analysis using CellProfiler 2.3 (53). DAPI channel was used for cell segmentation. Automatic identification of the RAD52 foci was done based on the intensity and size. At least 1000 cells were quantified in each condition. Statistical analysis was performed in GraphPad Prism 7.

### Statistical analysis

All statistical analyses were performed using GraphPad Prism 7.0 software. For the biochemical assays, Welch's t-test was used with *P* < 0.05 = *, *P* < 0.01 = **, *P* < 0.001 = ***, *P* < 0.0001 = ^****^. GFP-reporter assays were analyzed using two-way ANOVA test with *P* < 0.05 = *, *P* < 0.01 = **, *P* < 0.001 = ***, *P* < 0.0001 = ^****^.

## RESULTS

### DSS1 plays roles in both BRCA2- and RAD52-dependent DSB repair pathways

**Figure 1.**
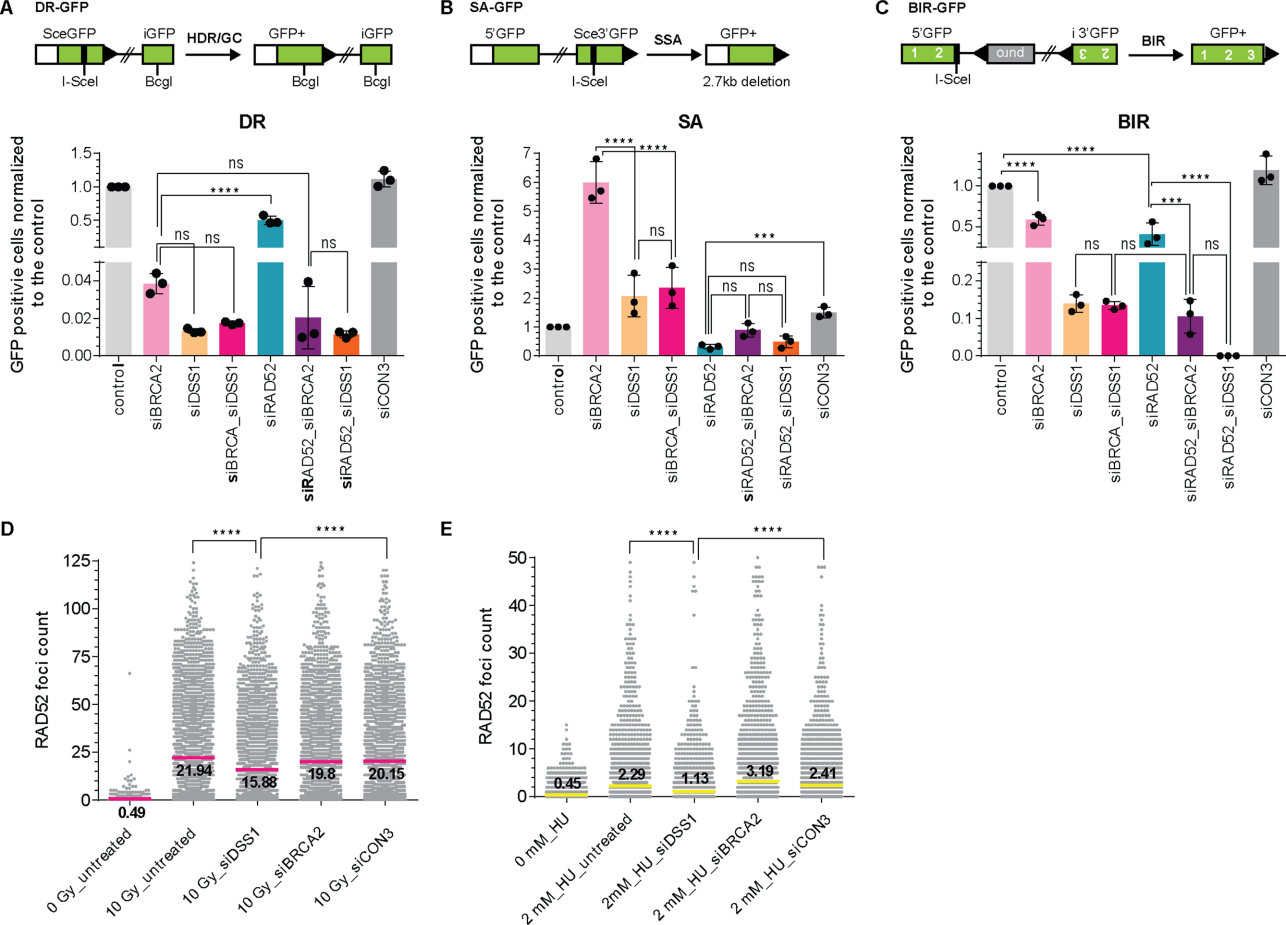
DSS1 affects various DSBs repair pathways and RAD52 foci formation. (**A**) Schematic representation and evaluation of U2OS-DR-GFP reporter assay normalized to the control sample that corresponds to 1.7% GFP+ cells. (**B**) Schematic representation and evaluation of U2OS-SA-GFP reporter assay normalized to the control sample that corresponds to 2.4% GFP+ cells. (**C**) Schematic representation and evaluation of U2OS-BIR-GFP reporter assay normalized to the control sample that corresponds to 1.7% GFP+ cells. The mean values (±SD) of data from three independent experiments were plotted and analysed using 2way ANOVA test (*P* < 0.001 = ***; *P* < 0.0001 = ^****^; ns not significant). (**D**) Quantification of YFP-RAD52 foci at 2 h after exposure to 10 Gy X-rays. The mean values of three independent experiments are shown, *P* < 0.0001 = ^****^ (**E**) Quantification of YFP-RAD52 foci at 4 h after treatment with 2 mM HU. The mean values of three independent experiments are shown, *P* < 0.0001 = ^****^.

To better understand the role of the human RAD52 protein in DSB repair and genome stability, we performed a series of I-SceI inducible GFP-reporter assays to test the efficiency of HR, SSA and break-induced replication (BIR) in BRCA2- or RAD52-depleted U2OS cell lines (Figure 1A–C). DSS1 plays an important role in BRCA2 stabilization (54) and cellular localization (40). Therefore, we also tested the effect of DSS1 depletion by siRNA on the HR, SSA and BIR in the presence and absence of BRCA2 and RAD52 (Supplementary Figure S1A). In the DR-GFP reporter assay we observe that BRCA2 is epistatic to DSS1 as expected supporting their shared function in BRCA2-repair events. Down-regulation of BRCA2 or DSS1 alone showed almost complete elimination of HR (app. 4% or 1% residual activity respectively) without further effect of their co-depletion. We also observe that RAD52 is epistatic to DSS1. Down-regulation of RAD52 exhibited a mild effect with 50% reduction of HR and its co-depletion with BRCA2 or DSS1 led to the decrease in HR efficiency comparable to single depletions of BRCA2 or DSS1 protein alone (Figure 1A). We have not observed any effect of individual siRNA on cell cycle phase distribution (Supplementary Figure S1B). Together, these data show that BRCA2-DSS1 are the primary recombination mediator while RAD52 plays a role to a lesser extent in the repair of DSBs.

Since the dysregulation of HR factors channels DSB repair to SSA (55), an alternative mechanism of DSBs repair in which RAD52 has an acknowledged role, we tested the effect of the same depletions (Supplementary Figure S1A) on SSA. We observe that BRCA2 is epistatic to DSS1 in the SSA-GFP reporter assay. While BRCA2-depleted cells showed a 6-fold stimulation of SSA, down-regulation of RAD52 resulted in more than a 3-fold decrease of SSA efficiency (Figure 1B). Interestingly, DSS1 depletion led to a small increase (<2-fold) of SSA efficiency and no additional effect was observed in BRCA2 co-depletion, indicating that DSS1 negates the effect of the BRCA2 absence and suggests a role of DSS1 in SSA pathway. These data suggest that in the absence of BRCA2, DSS1 helps RAD52 to deal with the resulting DSB burden channelled from non-functional HR pathway.

RAD52 was also reported to participate in BIR (24), thus we monitored the effect of RAD52, BRCA2 and DSS1 in the BIR-GFP reporter assays. We found that while BRCA2 was epistatic to DSS1, RAD52 was not epistatic to DSS1 in this assay. We observed that RAD52 depletion decreased the BIR efficiency by half, similarly to the depletion of BRCA2 (Figure 1C, S1A). Their co-depletion resulted in almost 10-fold decrease in BIR, indicating that they play partially overlapping roles in BIR. Intriguingly, depletion of DSS1 abrogated BIR to the same effect as RAD52/BRCA2 co-depletion, further supporting its role in both DSB repair pathways. Almost no BIR was detectable upon co-depletion of RAD52 and DSS1, indicating an unknown and overlapping role of the RAD52 and DSS1 proteins in BIR. In conclusion, these GFP-reporter experiments allowed us to determine that RAD52 and DSS1 function in the same pathway in HR and SSA.

As the depletion of DSS1 significantly influenced the efficiency of RAD52-dependent repair (SSA and BIR), we next monitored YFP-RAD52 foci formation by fluorescence microscopy following treatment with ionizing radiation (10 Gy). While no foci were formed in non-irradiated cells (Figure 1D, S1C), depletion of DSS1 led to a significant decrease in YFP-RAD52 foci formation, in contrast to BRCA2 depletion (Figures 1D, S1C). Next, we monitored RAD52 foci formation after replication stress in U2OS cells treated with (2 mM HU) for 4 h. Under these conditions, we observed YFP-RAD52 foci were formed less frequently when compared to IR induced YFP-RAD52 foci (Supplementary Figure S1C and D). However, the number of YFP-RAD52 foci was significantly reduced in DSS1-depleted U2OS cells (Figure 1E, S1D). Altogether, our cell-based data present for the first time evidence that DSS1 plays an important role not only in HR, but also in RAD52-dependent DNA repair pathways such as SSA and BIR.

### DSS1 directly binds to RAD52

**Figure 2.**
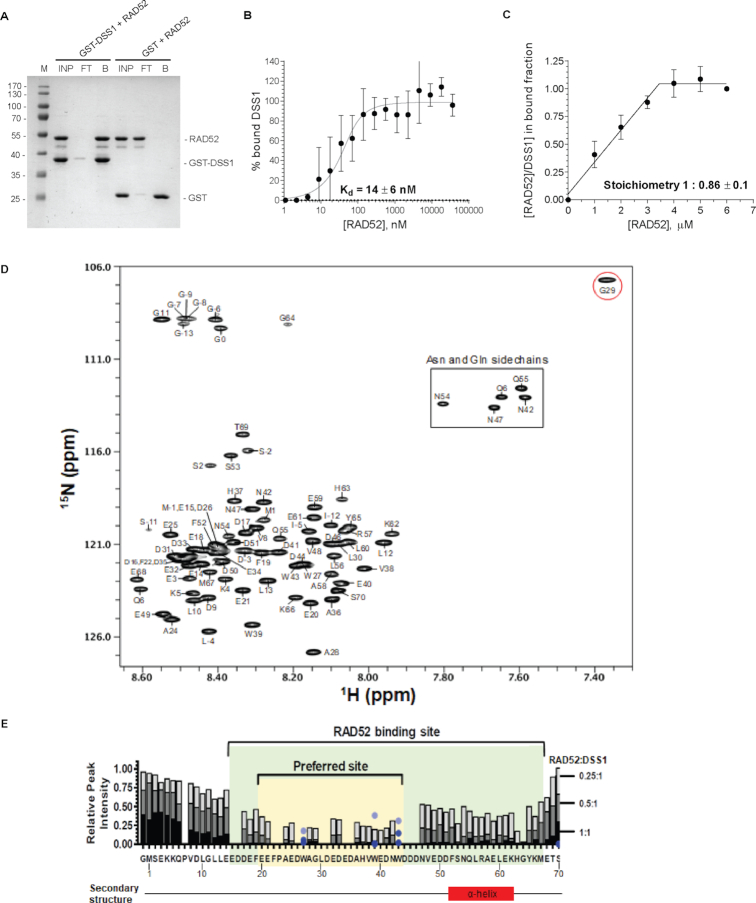
DSS1 interacts with RAD52 with high affinity and 1:1 stoichiometry. (**A**) GST pull-down assay with GST-DSS1 and RAD52 (5 μM each). The input (INP), the flow through (FT) and the bound (B) fractions were analysed. (**B**) Microscale Thermophoresis (MST) analysis of RAD52 interaction with DSS1. The data represent the average ± SD for two sets of three independent experiments. In each experiment the data were normalized to the average value of the last three data points set as 100% bound. Quadratic binding equation was used to calculate the *K*_d_. (**C**) Quantification of the pull-down experiments with GST-DSS1 immobilized on the beads and increasing concentration of RAD52. The data are shown as the average and standard deviation of three independent experiments. (**D**) Assigned ^15^N/^1^H HSQC spectrum of DSS1 with peaks labelled using DSS1 protein sequence numbering. The extra N-terminal tag (17 residues of GSPGISGGGGGILDSMG) in the protein construct are numbered as −16 to 0. The G29 amide peak circled in red exhibits unique chemical shift. (**E**) NMR-based analysis of the RAD52-DSS1 interaction. Overlaid histograms of the relative peak intensity of the assigned backbone amides of DSS1 (10 μM) in the ^15^N/^1^H HSQC spectra with bars coloured in light grey, medium grey, and dark for each residue of DSS1 for the samples containing 0.25:1, 0.5:1, and 1:1 RAD52:DSS1 ratios, respectively. For each assigned residue (including the tag-derived G0), the relative peak intensity was obtained by measuring the peak intensity of ^15^N-labelled DSS1 in the ^15^N/^1^H HSQC spectrum in the presence of RAD52 (at indicated ratios) divided by the peak intensity in the absence of RAD52. Relative peak intensity is not shown for the two prolines and thirteen other residues whose cross-peaks were severely overlapped in the ^15^N/^1^H HSQC spectra. The light blue, medium blue, and dark blue circles indicate the relative peak intensity of the tryptophan indole NϵH cross-peaks of W27, W39, and W43 of DSS1 for the samples containing 0.25:1, 0.5:1 and 1:1 RAD52:DSS1 ratios, respectively. The region of DSS1 involved in the RAD52 interaction (E15-M67) is shaded in green, the initial/preferred binding site (E20-W43) is shaded in yellow. Existence of an α-helix (F52-K62) as predicted from the assigned backbone chemical shifts of DSS1 in the absence of RAD52 using TALOS+ program is shown below the panel.

Next, we tested if recombinant GST-DSS1 protein could bind directly to the RAD52 protein *in vitro* by pull-down analysis. Indeed, GST-DSS1 was able to retain RAD52 on GTH-beads, compared to the control experiments (Figure 2A), confirming the novel interaction between these two proteins. As both proteins are evolutionary conserved in eukaryotes (31,56), we next tested for interaction of their yeast homologs (Rad52 and Sem1). Surprisingly, GST-tagged Sem1 did not show any interaction with yeast Rad52 (Supplementary Figure S2F), indicating that this interaction is not evolutionarily conserved. In contrast, GST-DSS1 was able to pull-down yeast Rad52 protein. Microscale thermophoresis (MST) also confirmed the interaction and allowed us to determine the binding affinity by mixing constant amount of Cy3-labelled DSS1 protein with an increasing concentration of RAD52 protein. Evaluation of the data revealed very strong interaction with a *K_d_* value of ∼14 nM (Figure 2B). Next, we analysed the stoichiometry of the RAD52–DSS1 interaction by a quantitative recombinant GST pull-down analysis where GST-DSS1 (4 μM) was incubated with increasing concentrations of RAD52 (Supplementary Figure S2E), indicating the 1:1 stoichiometry of GST-DSS1 to RAD52 (Figure 2C).

To understand mechanistically how DSS1 binds to RAD52, we performed binding assays in solution using NMR spectroscopy and mapped out the RAD52 interaction region on the DSS1 protein. To determine which amino acids in DSS1 bind to RAD52, we first made backbone and sidechain assignments of DSS1 by using a 220 μM uniformly [^15^N,^13^C]-labelled DSS1 protein (Figures 2D and S2A). These DSS1 assignments were deposited into the BMRB database with an accession number 27475. From these assignments, we found that while most of DSS1 lacks secondary structure, a region involving DSS1 residues F52-K62 form an α-helix in solution (Figures 2E and S2B). Then, we determined the regions of DSS1 which bind to RAD52 by performing binding assays through collecting a series of ^15^N/^1^H HSQC spectra of 10 μM ^15^N-labelled DSS1 in the absence and then in the presence of 2.5, 5 and 10 μM RAD52. The acquired data were then analysed by comparing peak heights that were normalized to the peak intensity in the absence of RAD52 and plotted as an overlaid bar graph as a function of DSS1 protein sequence (Figure 2E). At a 1:1 RAD52:DSS1 ratio (dark bar), RAD52 binding caused nearly complete peak broadening of the DSS1 backbone amides from E15 to M67, indicating that RAD52 binds to E15-M67 of DSS1 tightly, consistent with the MST binding data. At 0.25:1 RAD52:DSS1 ratio (light grey bar), E20 to W43 of DSS1 are more selectively broadened by the binding of RAD52, indicating that these DSS1 residues are the preferred binding site for RAD52. Interestingly, the α-helix (F52-K62) present in DSS1 in solution (Figure 2E and S2B) forms a part of the RAD52-binding site on DSS1, while the N-terminal residues (M1-E14) and the last three C-terminal residues of DSS1 are not involved in RAD52 binding. Moreover, the α-helical structure is consistent with a much larger ^15^N{^1^H} heteronuclear NOE (Supplementary Figure S2C) and a reduced ^15^N T_2_ relaxation time (Supplementary Figure S2D) for these DSS1 helical residues, likely due to the restricted motion or ordered structure of this α-helix compared to the rest of the DSS1 residues. Furthermore, the DSS1 residue G29, located in the RAD52-preferred binding site (E20-W43), exhibits a unique chemical shift (Figure 2D), suggesting the existence of a distinct protein conformation around residue G29. As the RAD52-binding region on DSS1 (E15-M67) is highly acidic and contains seven aromatic residues (F19, F22, W27, W39, W43, F52, Y65), it is highly likely that DSS1 binding to RAD52 involves ionic interactions between the negatively charged residues of DSS1 and positively charged residues of RAD52 as well as hydrophobic interactions between them.

We have also used EM to analyse the effect of DSS1 on RAD52 structure, however under buffer conditions identical to biochemical assays we observed very heterogenous and dynamic behaviour with no clear symmetry that prevented us from 3D model reconstruction. Nevertheless, 2D class averages of RAD52 and its complex with DSS1 and/or DNA suggest conformation changes upon DSS1 binding (Supplementary Figure S3). Taken together, we show that the human DSS1 protein contains an ordered α-helix (F52-K62) in solution and that this helix is involved in binding to the RAD52. We also show that the DSS1 protein is a new high affinity RAD52 interacting partner with 1:1 binding stoichiometry, inducing conformational change within RAD52, and requires E20 to W43 of the DSS1 for the binding.

### RAD52 biochemical activities are modulated by DSS1

**Figure 3.**
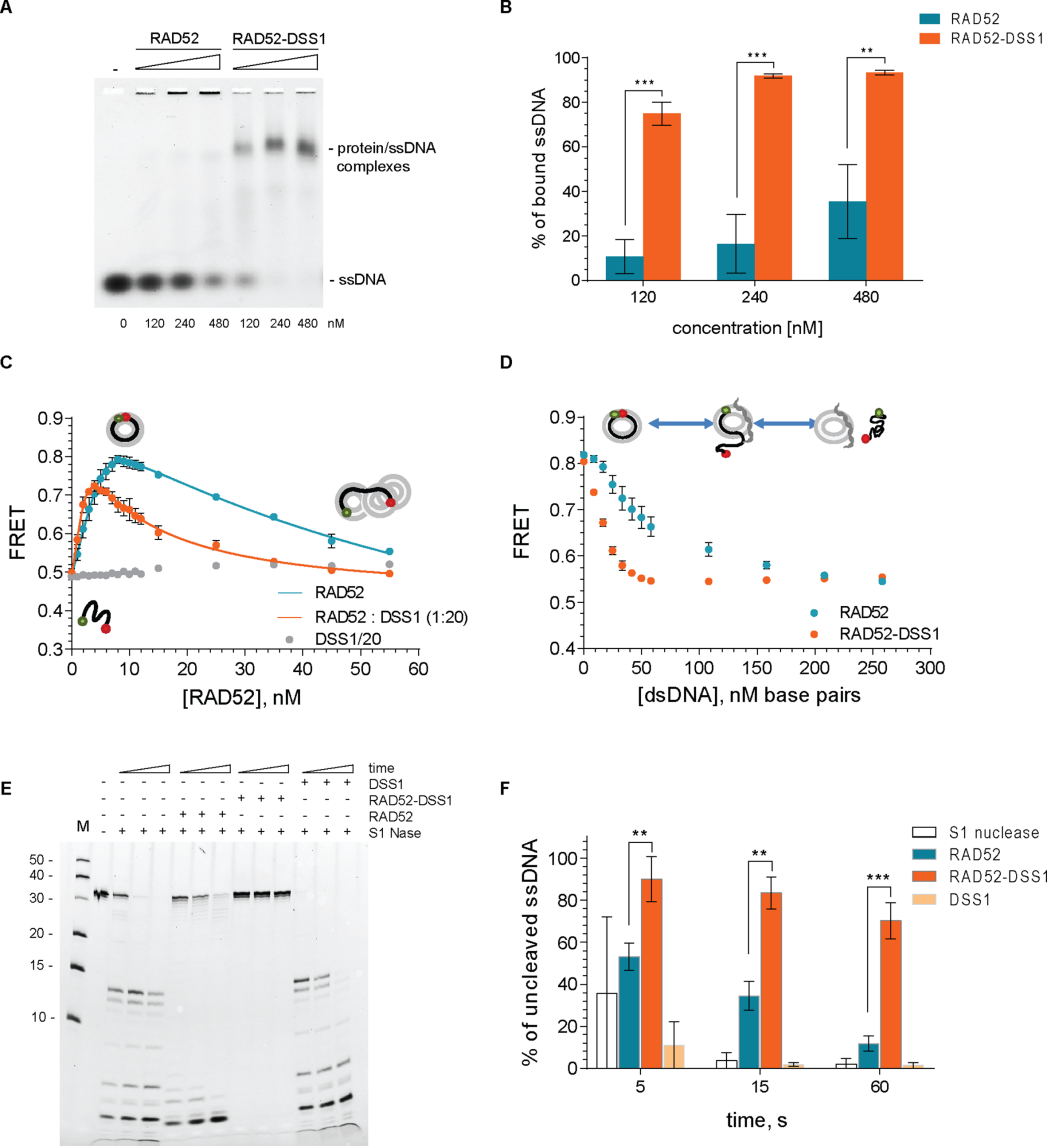
DSS1 affects the mode of the ssDNA wrapping around the RAD52 oligomeric ring. (**A**) EMSA with fluorescently labelled 33-mer ssDNA incubated with increasing concentration of RAD52 or preformed RAD52-DSS1 complex. Protein–ssDNA complexes and free ssDNA are indicated. (**B**) Quantification of three independent EMSA assays. Mean values ± SD were plotted (Welch's *t*-test, *P* < 0.01 = **; *P* < 0.001 = ***). (**C**) FRET-based binding ssDNA binding assay for RAD52 or RAD52-DSS1 (1:20) respectively. The ssDNA–RAD52 interaction changes the FRET between Cy3 and Cy5 dyes. Each data point represents average and standard deviation from three independent experiments. Continues line represent fitting of the data to two-step binding isotherm. (**D**) FRET-based dsDNA competition assay using linearized plasmid DNA. FRET signal decreases as ssDNA is released from RAD52 or RAD52-DSS1 due to binding of the dsDNA. The mean values ± SD from three independent experiments are plotted. (**E**) S1 nuclease protection assay in the presence of RAD52 or RAD52-DSS1 complex. (**F**) Evaluation of the percentage of uncleaved ssDNA substrate from three independent experiments using Welch's *t*-test, *P* < 0.01 = **; *P* < 0.001 = ***.

Next, we tested the effect of DSS1 on RAD52-mediated activities monitoring ssDNA binding by an electrophoretic mobility shift assay (EMSA). The pre-formed RAD52-DSS1 complex was able to bind ssDNA (15 nM) with higher affinity compared to the RAD52 alone (Figure 3A). Specifically, while RAD52 alone at 240 nM shows less than 20% binding of ssDNA, over 80% of ssDNA is bound by 240 nM in the RAD52–DSS1 complex (Figure 3B). Moreover, the ssDNA–RAD52–DSS1 complexes were observed as discrete bands with faster mobility in contrast to the ssDNA–RAD52 complexes indicating that the DSS1 interaction increases the mobility of the ssDNA–RAD52 complex leading to more defined engagement with ssDNA. To further confirm this effect, we utilized previously described FRET-based assays that monitor the ability of RAD52 to bind and wrap ssDNA around the narrow groove spanning the circumference of the protein ring (50). FRET donor (Cy3) and acceptor (Cy5) fluorophores were positioned at the ends of a 30-mer ssDNA (Cy3-dT30-Cy5). The binding and wrapping of Cy3-dT30-Cy5 around the RAD52 ring bring the two fluorophores in close proximity resulting in an increase in FRET (Figure 3C). The maximum intensity was observed at ratio corresponding to 1 oligonucleotide per 7–8 RAD52 monomers. Further, addition of RAD52 resulted in a decrease in the FRET signal likely due to binding of the ssDNA within multiple RAD52 rings. The biphasic shape of the binding curve reflects two different geometries of the ssDNA, fully wrapped around one ring (high FRET) and shared between multiple RAD52 rings (low FRET at high RAD52 concentrations) with two distinct binding affinities (*K*_*d1*_ and *K*_*d2*_, respectively) (50). In the reaction with increasing concentration of pre-formed RAD52 and DSS1 complex at saturating concentration of DSS1 (1:20), we observed three significant changes in the binding curve (Figure 3C). First, the maximum FRET signal was reduced from about 0.8 to 0.7. Second, the concentration of RAD52 at the maximum FRET value was two times lower when bound by DSS1. Finally, we observe decreased FRET values at higher RAD52 concentrations. All three changes in the binding isotherm may stem from the change in the binding stoichiometry (*n*_(RAD52)_ = 5.5 ± 0.5 and *n*_(RAD52-DSS1)_ = 2.5 ± 0.3 monomers per 30-mer oligo ssDNA) combined with the increased affinity of the second binding mode (the *K_d2_* is about 14 nM for RAD52 alone and is about 4 nM for the RAD52-DSS1). Interestingly, this phenomenon is ssDNA specific since we only observed very small changes in the binding of dsDNA by RAD52 compared to RAD52-DSS1 complex using EMSA and FRET-based dsDNA binding methods (Supplementary Figure S4A–C).

To further compare the differences in RAD52 and RAD52–DSS1 ssDNA binding, we used a stopped flow approach previously used to monitor RAD51 binding to ssDNA (57). Cy3 labelled dT33-mer ssDNA was quickly mixed with increasing concentrations of RAD52 or RAD52-DSS1 pre-formed complex and the time dependence of binding was measured by following the change in Cy3 fluorescence (Figures S4D, E). Evaluation of the stopped flow data shows significant differences between RAD52 and RAD52–DSS1 binding to ssDNA. First, RAD52 binding to ssDNA leads to smaller fluorescence amplitude compared to RAD52-DSS1, correlating to lower affinity to ssDNA (Figures S4F). Second, tested proteins also show a change of values from negative to positive and differ in the breakpoint between the values of fluorescence change. These changes could be accounted for possible structural rearrangement or represent the concentration where one ssDNA molecule is bound between more than one ring. Specifically, while RAD52 binding to DNA results in decrease of the fluorescence signal, binding of RAD52-DSS1 complex to DNA show increased signal. Furthermore, this change of fluorescence increases for RAD52–DSS1 to positive values between the concentrations of 0.1–0.25 μM. In case of RAD52, this point is shifted to higher concentration range (∼0.5 μM, Supplementary Figure S4F), further supporting the DNA binding-induced changes on RAD52 in the presence of DSS1.

### DSS1 alters engagement of RAD52 with ssDNA

To monitor the DNA accessibility by nuclease protection assay using S1 nuclease, a single-strand-specific endonuclease we pre-incubated RAD52 or RAD52–DSS1 complex with ssDNA and treated with S1 nuclease (0.2 U) in time course. The analysis indicates that ssDNA bound by RAD52 ring becomes completely degraded at 1 hour, while the RAD52–DSS1 complex shows almost full protection of the ssDNA from the nuclease degradation (Figure 3E, F). This suggests that a conformation change occurs in the RAD52–DSS1 complex leading to ssDNA protection. To assess if RAD52 dsDNA binding is also modulated in the presence of DSS1 we performed FRET-based competition assays (58) where RAD52 (8 nM) or RAD52–DSS1 (8 nM) bound to Cy3-dT30-Cy5 (1 nM) was challenged with linearized dsDNA (ΦX174 RF I DNA digested with ApaLI). Titration of RAD52 (8 nM) bound to Cy3-dT30-Cy5 (1 nM) with linearized dsDNA resulted in ssDNA release as monitored by a decrease in FRET signal (Figure 3D), with nearly all ssDNA released at 200 nM base pairs of dsDNA. Similar experiment with the RAD52-DSS1 complex displayed almost complete ssDNA release already at 50 nM base pairs of linearized dsDNA, suggesting that DSS1 enhances four-fold the release of ssDNA when challenged with dsDNA. These data were confirmed by measurements of the dsDNA competition using stopped flow, which allows us to observe the ssDNA displacement in time (Supplementary Figure S4G). RAD52 or RAD52–DSS1 complex was mixed with ssDNA and then the reaction was quickly mixed with an excess of dsDNA while monitoring the changes in fluorescence. The presence of dsDNA led to the decrease in fluorescence signal in both RAD52–ssDNA and RAD52–DSS1–ssDNA samples. Again, this change was more evident in the case of RAD52–DSS1–ssDNA complex (Supplementary Figure S4G, H).

### DSS1 stimulates RAD52-mediated annealing

**Figure 4.**
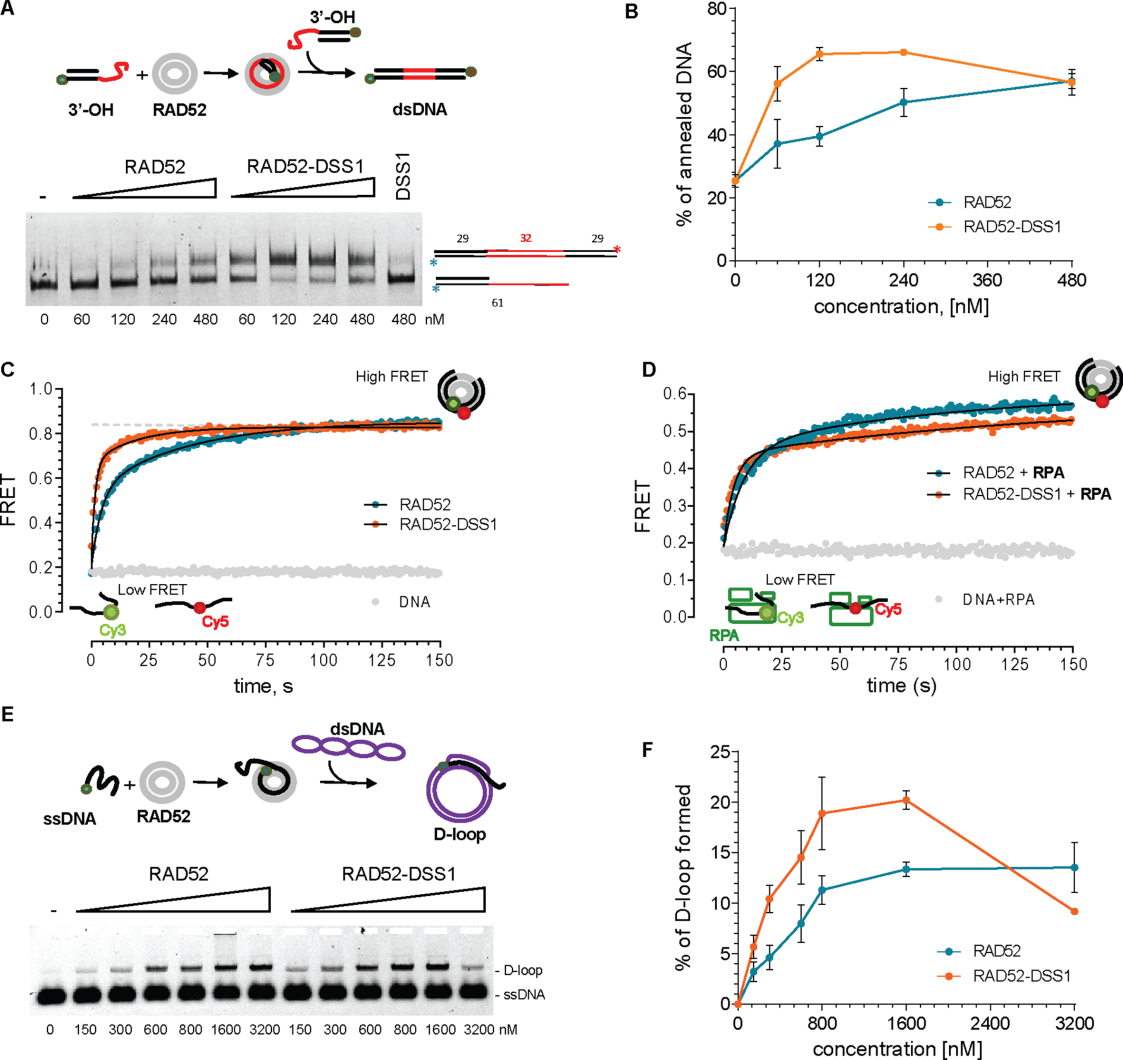
RAD52 annealing and homology search are stimulated by DSS1. (**A**) Gel-based SSA assay using complementary fluorescent 3′-OH substrates in the presence of indicated concentration of RAD52 or RAD52–DSS1 complex. The position of substrate and product of annealing reaction are indicated. (**B**) Quantification of the annealing reactions from three independent experiments. The mean values ± SD were plotted. (**C**) FRET based ssDNA annealing experiments of complementary 28-mer oligonucleotides labelled with Cy3 and Cy5 dyes, respectively in the presence of RAD52 (8 nM, blue) and RAD52-DSS1 complex (8 nM-40 nM, orange). (**D**) FRET based ssDNA annealing experiment of complementary 28-mer oligonucleotides labelled with Cy3 and Cy5 dyes in the presence of RPA (2 nM), respectively in the presence of stoichiometric amounts of RAD52-RPA (8–2 nM, blue) and RAD52-RPA-DSS1 (8–2–40 nM, orange) complex. These data are fit to a double exponential (solid lines). (**E**) Scheme of *in vitro* D-loop reaction and representative agarose gel showing the formation of D-loop using indicated amounts of RAD52 or RAD52–DSS1. (**F**) Quantification of the D-loop reaction from three independent experiments. The mean values ± SD from were plotted.

Since RAD52 and DSS1 were both observed to play a role in SSA pathway, we next assessed how DSS1 modulated RAD52 annealing activity. In a standard gel-based annealing reaction, increasing concentration of RAD52 or RAD52–DSS1 was pre-equilibrated with saturating amounts of overhang DNA substrates with 32 nts complementary regions (Figure 4A). The analysis of the products revealed optimum of the reaction at 120 nM RAD52–DSS1 complex in contrast to 480 nM of RAD52, suggesting that DSS1 promotes the efficiency of the reaction four-fold. Moreover, the maximum yield was also different, showing 50% and 65% for RAD52 and RAD52–DSS1, respectively (Figure 4B).

To confirm our conclusions from gel-based SSA assay and to study the observed SSA stimulation by DSS1 in time, we performed FRET-based annealing assays with RAD52 or RAD52–DSS1 (8 nM RAD52, 40 nM DSS1 and 0.5 nM 28 nucleotide substrates) in the absence (Figure 4C) and presence of 2 nM RPA (Figure 4D). Under these concentrations RPA saturates the substrate. The kinetic parameters of the reactions (Supplementary Table S5) and the calculated initial rates for the RAD52 and RAD52–DSS1 complex (Supplementary Table S6) show that DSS1 stimulates the annealing activity of RAD52 in the absence (from 5.2 bp/s to 17.5 bp/s) as well as presence of RPA (5.7–12 bp/s). Taken together, our results show that DSS1 stimulates the initial rate of RAD52 ssDNA annealing both in the absence or presence of RPA.

### RAD52 is able to form a D-loop and DSS1 enhances this activity

To test whether RAD52 can promote D-loop formation, a function that would be required in the BIR pathway, we checked its ability to form D-loops using a 90-mer ssDNA oligonucleotide that is complementary to a negatively supercoiled plasmid. RAD52 or RAD52–DSS1 were first pre-incubated with ssDNA and then the plasmid DNA was added to the reaction. Formation of the D-loop structure was observed as an appearance of slowly migrating band in agarose gel that was comparable to the product of the RAD51 mediated D-loop formation (Supplementary Figure S5). RAD52 shows D-loop formation in a concentration-dependent manner reaching more than 10% yield (Figure 4E, F). The RAD52–DSS1 complex was able to form the D-loop even more efficiently with almost 20% yield observed at a concentration of 800 nM of RAD52–DSS1, compared to 10% for RAD52 alone (Figure 4E, F). Similar to annealing, the presence of DSS1 in the reaction decreased the amount of RAD52 required to achieve the same extent of D-loop formation. Specifically, to reach 15% D-loop efficiency four times more RAD52 protein is required compared to the complex with DSS1 (Figure 4F). These results demonstrate the ability of RAD52 to form D-loops providing key activity for RAD51-independent BIR pathway. Furthermore, this activity is stimulated by the DSS1 protein correspondingly to the phenotypes observed in our GFP-reporter BIR assays.

## DISCUSSION

Exploitation of the synthetic lethal relationship between defects in BRCA1 and BRCA2 tumour suppressors and with RAD52 depletion or inhibition (15–18,20,59,60) has opened many new questions into the role of the RAD52 protein in genome maintenance. As the depletion of RAD52 in mammalian cells did not show any severe phenotypes initially (21), for decades it was believed that the role of RAD52 was only minor with BRCA2 serving as the main recombination mediator for HR. During a protein-protein interaction screen among varying HR proteins, we observed that the small and highly acidic protein DSS1 interacts with the RAD52 protein. This was an interesting discovery, since DSS1 was previously identified as an interacting partner of BRCA2 that is necessary for the proper activity of this main mammalian recombination mediator protein (39,40,61). Therefore, we aimed to explore the role of the DSS1 interaction with RAD52 protein. We found that RAD52 foci are perturbed when DSS1 is depleted in cells treated with IR and HU. To determine how complex formation between RAD52 and DSS1 affects repair outcomes in mammalian cells, we utilized the I-SceI inducible GFP-reporter assays (51,52). Not surprisingly, BRCA2 depletion increased SSA indicating that the DSB load is being redirected towards SSA pathway. On the other hand, DSS1 depletion, despite similar HR inactivation as the absence of BRCA2, shows only a small SSA increase indicating that it likely helps RAD52 to deal with the DSB burden corroborating direct role of DSS1 in this pathway. This is further supported by the suppression of SSA levels in co-depletion of DSS1 with BRCA2 and the epistatic relationship with RAD52 co-depletion. Surprisingly, we observed the strongest defects from DSS1 depletion in the BIR reporter assay. Co-depletion of RAD52 and DSS1 show an even further decrease of BIR efficiency compared to the absence of DSS1 alone or BRCA2/RAD52 co-depletion. This could be due to the versatility of the DSS1 interactome, including the recently described interaction of DSS1 with RPA and mediation of the ssDNA handoff with other proteins within its interactome (41,62). We thus conclude that DSS1 is involved in both BRCA2/RAD51-dependent and -independent branches of DSB repair.

To understand mechanistically how DSS1 binds to RAD52 and affects RAD52 function we performed a biochemical analysis. We found that DSS1 binds RAD52 with 1:1 stoichiometry with very high affinity (*K*_d_ ∼ 14 nM). Our NMR studies also revealed a tight interaction between DSS1 and RAD52 involving almost the entire DSS1 length from E15 to M67, with the initial preferred binding site in the region E20-W43 of DSS1 and confirmed the presence of an α-helix from F52-K62 near DSS1 C-terminus (63). Interestingly, despite the relatively high degree of identity (47%) and similarity (69%) between human DSS1 and its yeast homolog Sem1 (56), the RAD52–DSS1 interaction is not conserved in yeast. RAD52 was very recently identified in the DSS1 interactome in *S. pombe* (62), which together with our data opens a question whether the DSS1 protein evolved to serve as a new tool for regulation of conserved, yet not essential RAD52 protein to help promote and coordinate DSB repair in humans. We also performed structural analysis using EM that suggest conformational changes of the RAD52 ring upon binding DSS1 and short single-stranded DNA. However, observed structural heterogeneity, likely due to the dynamic nature of this complex, prevented us from successful 3D classification and auto-refinement, but indicate lack of clear symmetry under the same conditions used for the biochemical studies.

To assess if DSS1 modulates the ssDNA and dsDNA binding activities of RAD52 several assays were performed. FRET-based biophysical ssDNA binding assays comparing RAD52 and RAD52-DSS1 complexes show DSS1 enhances the binding affinity of the RAD52 ring for ssDNA. This may be due to the ssDNA binding region occupying a larger physical space allowing accommodation of multiple DNA molecules or stacking within the ssDNA and dsDNA grooves simultaneously. FRET-based dsDNA competition assays also suggest the shape of the RAD52–DSS1 ring is structurally different than RAD52 alone. Stopped flow experiments and S1 nuclease protection assays support a more complex behaviour in the presence of DSS1. DSS1 also stimulated the annealing rate of RAD52 in gel-based and FRET-based assays. Importantly, this was also observed on RPA-coated ssDNA representing more physiological substrate. These data are in agreement with the proposed *trans* mechanism for RAD52-mediated annealing, where the most efficient search for homology and consequent annealing requires two RAD52 complexes and release of ssDNA from RAD52 followed by dsDNA zippering occurs via successive rearrangement of these complexes (38,43). Alternatively, faster or more specific ssDNA binding within the inner RAD52 binding site could promote base pairing by faster movement of the paired DNA to the outer binding site of the RAD52 rings. Another possible explanation for these phenomena could be that DSS1 binding on RAD52 ring could lead to the switch from a *trans* to *cis* SSA mechanism in which the complementary ssDNA strands bind and anneal on a single RAD52 ring (37).

**Figure 5.**
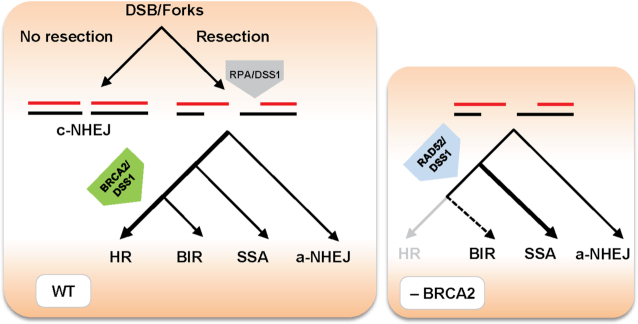
Model describing possible mechanism of RAD52-DSS1 activity in the DSB repair. DSBs or stalled replication forks are resected to produce ssDNA required for homology-directed repairs, alternatively blocked resection allows repair by c-NHEJ. While initial small resection makes DNA ends available for a-NHEJ, extensive resection generates long ssDNA stretches occupied by RPA. BRCA2 in the complex with DSS1 helps to alleviate RPA inhibitory effect and load RAD51 presynaptic filament to promote HR. RAD52-mediated SSA/BIR represents an alternative repair pathway, where DSS1 may facilitate this process via stimulation of RAD52 binding and strand invasion activities. Therefore, in the absence of BRCA2, RAD52/DSS1 becomes essential to process breaks/forks via SSA/BIR pathways.

Combining our results with previous findings, we propose a model (Figure 5), where DSBs or stalled replication forks are resected by the MRE11 nuclease to produce ssDNA required for homology-directed repair. Resection generates long ssDNA stretches occupied by RPA (64), blocking the ssDNA accessible for a-NHEJ (65) and promoting recombination repair. In HR, BRCA2 ensures the repair through loading of the RAD51 presynaptic filament and helps alleviate the RPA inhibitory effect. DSS1 bound to BRCA2 and RPA facilitates handoff of ssDNA from RPA to RAD51. RAD52-mediated SSA/BIR represents an alternative DSB repair pathway, where DSS1 seems to facilitate this process via stimulation of the RAD52 binding and D-loop formation activities. However, RAD52 might promote strand exchange reaction by different mechanism then RAD51 or RecA based on its ability to change dsDNA structure, intercalating into the helix to allow base pairing (66). Therefore, in the absence of BRCA2, RAD52-DSS1 becomes essential to repair processed breaks or forks via SSA/BIR pathways.

The precise mechanism of how RAD52 and DSS1 act in SSA and BIR pathways will require further studies. Several pieces of evidence point out to their important role in these processes, especially in the absence of functional BRCA2 protein. Cancer inactivating BRCA2 mutations are characterized by large tandem duplications and deletions (67) which are typical for RAD52-mediated BIR and SSA repair mechanisms, contributing to rearrangements, fuelling genomic instability and oncogenic transformation (24,68). Similar results were obtained for ATM- and POLQ-deficient mice (69), suggesting that POLQ or RAD52 is predominantly used for DNA repair upon a HR deficiency. This is likely the case as RAD52 and POLQ have been shown to play a role in replication fork restart and at DSB sites >50 nucleotides (28). Cells rely on RAD52 also when DNA damage overloads the capacity of BRCA1 and 53BP1 (55) which could explain why the RAD52 gene is amplified in human cancers, and why its inactivation curtails cancer development (16,70,71). The absence of both BRCA2 and RAD52 likely results in utilization of resected DSB by a-NHEJ, further fuelling genomic instability. This has been reported in RAD52-dependent progression of pre-malignancy to squamous cell carcinoma where RAD52 depletion appeared to increase genomic instability beyond a manageable threshold acceding the cells to death rather than tumorigenesis (72). In addition, breast cancer samples with low RAD52 expression had more insertions/deletions and fusions (26), supporting the ability of RAD52 to suppress a-NHEJ pathway. Furthermore, DSS1 depletion also rendered breast and myeloma cells sensitive to DNA damage and reduction of copy number alterations (73), where BIR is implicated among the key pathways in their generation (74). Finally, analysis of DNA repair in p53-independent p21^WAF1/Cip1^ cells showed that upregulated RAD52 mediated mutagenic DSB repair via SSA and BIR (75). It would be interesting to know if increased levels of DSS1 would phenocopy this behavior.

Taken together we identified a new DSS1-mediated interaction with another DSB repair factor, RAD52. DSS1 modulates RAD52 activities and adds another mechanistic level of regulation when normal error free repair proteins are not present. The main conclusion from our model is that DSS1-RAD52 interaction could allow rescue for BRCA2-deficiency in cancer cells by promoting RAD52-mediated activities via SSA and BIR pathways. Utilization of various repair pathways corresponds to different genome instability signatures, a hallmark of most cancers, therefore reliance on RAD52–DSS1 could represent a promising therapeutic target for killing defined cancers.

## DATA AVAILABILITY

All the relevant data are available from the authors upon request.

## ACCESSION NUMBERS

Backbone and sidechain NMR assignments of DSS1 are deposited in the BMRB database with an accession number 27475.

## Supplementary Material

gkz1052_Supplemental_File

## Notes

Present address: Sarah R. Hengel, Department of Microbiology and Molecular Genetics, UPMC Hillman Cancer Center, University of Pittsburgh School of Medicine, 5117 Centre Avenue, Pittsburgh, PA 15213, USA.

## References

[1] Hanahan D., Weinberg R.A. Hallmarks of cancer: the next generation. Cell. 2011; 144:646–674. 21376230 10.1016/j.cell.2011.02.013

[2] Franchitto A., Pichierri P. Replication fork recovery and regulation of common fragile sites stability. Cell Mol. Life Sci. 2014; 71:4507–4517. 25216703 10.1007/s00018-014-1718-9 PMC11113654

[3] Kolinjivadi A.M., Sannino V., de Antoni A., Techer H., Baldi G., Costanzo V. Moonlighting at replication forks - a new life for homologous recombination proteins BRCA1, BRCA2 and RAD51. FEBS Lett. 2017; 591:1083–1100. 28079255 10.1002/1873-3468.12556

[4] Pasero P., Vindigni A. Nucleases acting at stalled forks: how to reboot the replication program with a few shortcuts. Annu. Rev. Genet. 2017; 51:477–499. 29178820 10.1146/annurev-genet-120116-024745

[5] Feng W., Jasin M. Homologous recombination and replication fork protection: BRCA2 and more!. Cold Spring Harb. Symp. Quant. Biol. 2017; 82:329–338. 29686033 10.1101/sqb.2017.82.035006 PMC6333483

[6] Symington L. Microbiology and Molecular Biology Reviews. 2002; 66:630–670. 12456786 10.1128/MMBR.66.4.630-670.2002 PMC134659

[7] New J.H., Sugiyama T., Zaitseva E., Kowalczykowski S.C. Rad52 protein stimulates DNA strand exchange by Rad51 and replication protein A. Nature. 1998; 391:407–410. 9450760 10.1038/34950

[8] Sung P. Function of yeast Rad52 protein as a mediator between replication protein A and the Rad51 recombinase. J. Biol. Chem. 1997; 272:28194–28197. 9353267 10.1074/jbc.272.45.28194

[9] Tsuzuki T., Fujii Y., Sakumi K., Tominaga Y., Nakao K., Sekiguchi M., Matsushiro A., Yoshimura Y., Morita T Targeted disruption of the Rad51 gene leads to lethality in embryonic mice. Proc. Natl. Acad. Sci. U.S.A. 1996; 93:6236–6240. 8692798 10.1073/pnas.93.13.6236 PMC39005

[10] Yuan S.S., Lee S.Y., Chen G., Song M., Tomlinson G.E., Lee E.Y. BRCA2 is required for ionizing radiation-induced assembly of Rad51 complex in vivo. Cancer Res. 1999; 59:3547–3551. 10446958

[11] Zellweger R., Dalcher D., Mutreja K., Berti M., Schmid J.A., Herrador R., Vindigni A., Lopes M. Rad51-mediated replication fork reversal is a global response to genotoxic treatments in human cells. J. Cell Biol. 2015; 208:563–579. 25733714 10.1083/jcb.201406099 PMC4347635

[12] Kolinjivadi A.M., Sannino V., De Antoni A., Zadorozhny K., Kilkenny M., Técher H., Baldi G., Shen R., Ciccia A., Pellegrini L. et al. Smarcal1-mediated fork reversal triggers mre11-dependent degradation of nascent DNA in the absence of Brca2 and stable Rad51 nucleofilaments. Mol. Cell. 2017; 67:867–881. 28757209 10.1016/j.molcel.2017.07.001 PMC5594205

[13] McIlwraith M.J., West S.C. DNA repair synthesis facilitates RAD52-mediated second-end capture during DSB repair. Mol. Cell. 2008; 29:510–516. 18313388 10.1016/j.molcel.2007.11.037

[14] Reddy G., Golub E.I., Radding C.M. Human Rad52 protein promotes single-strand DNA annealing followed by branch migration. Mutat. Res. 1997; 377:53–59. 9219578 10.1016/s0027-5107(97)00057-2

[15] Feng Z., Scott S.P., Bussen W., Sharma G.G., Guo G., Pandita T.K., Powell S.N. Rad52 inactivation is synthetically lethal with BRCA2 deficiency. Proc. Natl. Acad. Sci. U.S.A. 2011; 108:686–691. 21148102 10.1073/pnas.1010959107 PMC3021033

[16] Cramer-Morales K., Nieborowska-Skorska M., Scheibner K., Padget M., Irvine D.A., Sliwinski T., Haas K., Lee J., Geng H., Roy D. et al. Personalized synthetic lethality induced by targeting RAD52 in leukemias identified by gene mutation and expression profile. Blood. 2013; 122:1293–1304. 23836560 10.1182/blood-2013-05-501072 PMC3744994

[17] Hengel S.R., Malacaria E., Folly da Silva Constantino L., Bain F.E., Diaz A., Koch B.G., Yu L., Wu M., Pichierri P., Spies M.A. et al. Small-molecule inhibitors identify the RAD52-ssDNA interaction as critical for recovery from replication stress and for survival of BRCA2 deficient cells. Elife. 2016; 5:e14740. 27434671 10.7554/eLife.14740 PMC4982760

[18] Chandramouly G., McDevitt S., Sullivan K., Kent T., Luz A., Glickman J.F., Andrake M., Skorski T., Pomerantz R.T. Small-molecule disruption of RAD52 rings as a mechanism for precision medicine in BRCA-deficient cancers. Chem. Biol. 2015; 22:1491–1504. 26548611 10.1016/j.chembiol.2015.10.003 PMC4701204

[19] Huang F., Goyal N., Sullivan K., Hanamshet K., Patel M., Mazina O.M., Wang C.X., An W.F., Spoonamore J., Metkar S. et al. Targeting BRCA1- and BRCA2-deficient cells with RAD52 small molecule inhibitors. Nucleic Acids Res. 2016; 44:4189–4199. 26873923 10.1093/nar/gkw087 PMC4872086

[20] Lok B.H., Carley A.C., Tchang B., Powell S.N. RAD52 inactivation is synthetically lethal with deficiencies in BRCA1 and PALB2 in addition to BRCA2 through RAD51-mediated homologous recombination. Oncogene. 2012; 32:3552–3558. 22964643 10.1038/onc.2012.391 PMC5730454

[21] Rijkers T., Van Den Ouweland J., Morolli B., Rolink A.G., Baarends W.M., Van Sloun P.P., Lohman P.H., Pastink A. Targeted inactivation of mouse RAD52 reduces homologous recombination but not resistance to ionizing radiation. Mol. Cell Biol. 1998; 18:6423–6429. 9774658 10.1128/mcb.18.11.6423 PMC109228

[22] Yamaguchi-Iwai Y., Sonoda E., Buerstedde J.M., Bezzubova O., Morrison C., Takata M., Shinohara A., Takeda S. Homologous recombination, but not DNA repair, is reduced in vertebrate cells deficient in RAD52. Mol. Cell Biol. 1998; 18:6430–6435. 9774659 10.1128/mcb.18.11.6430 PMC109229

[23] Murfuni I., Basile G., Subramanyam S., Malacaria E., Bignami M., Spies M., Franchitto A., Pichierri P. Survival of the replication checkpoint deficient cells requires MUS81-RAD52 function. PLos Genet. 2013; 9:e1003910. 24204313 10.1371/journal.pgen.1003910 PMC3814295

[24] Sotiriou S.K., Kamileri I., Lugli N., Evangelou K., Da-Ré C., Huber F., Padayachy L., Tardy S., Nicati N.L., Barriot S. et al. Mammalian RAD52 functions in break-induced replication repair of collapsed DNA replication forks. Mol. Cell. 2016; 64:1127–1134. 27984746 10.1016/j.molcel.2016.10.038 PMC5179496

[25] Bhowmick R., Minocherhomji S., Hickson I.D. RAD52 facilitates mitotic DNA synthesis following replication stress. Mol. Cell. 2016; 64:1117–1126. 27984745 10.1016/j.molcel.2016.10.037

[26] Yasuhara T., Kato R., Hagiwara Y., Shiotani B., Yamauchi M., Nakada S., Shibata A., Miyagawa K. Human Rad52 promotes XPG-mediated r-loop processing to initiate transcription-associated homologous recombination Repair. Cell. 2018; 175:558–570. 30245011 10.1016/j.cell.2018.08.056

[27] Malacaria E., Pugliese G.M., Honda M., Marabitti V., Aiello F.A., Spies M., Franchitto A., Pichierri P. Rad52 prevents excessive replication fork reversal and protects from nascent strand degradation. Nat. Commun. 2019; 10:1412. 30926821 10.1038/s41467-019-09196-9 PMC6441034

[28] Kelso A.A., Lopezcolorado F.W., Bhargava R., Stark J.M. Distinct roles of RAD52 and POLQ in chromosomal break repair and replication stress response. PLoS Genet. 2019; 15:e1008319. 31381562 10.1371/journal.pgen.1008319 PMC6695211

[29] Stasiak A.Z., Larquet E., Stasiak A., Müller S., Engel A., Van Dyck E., West S.C., Egelman E.H. The human Rad52 protein exists as a heptameric ring. Curr. Biol. 2000; 10:337–340. 10744977 10.1016/s0960-9822(00)00385-7

[30] Van Dyck E., Hajibagheri N.M., Stasiak A., West S.C. Visualisation of human rad52 protein and its complexes with hRad51 and DNA. J. Mol. Biol. 1998; 284:1027–1038. 9837724 10.1006/jmbi.1998.2203

[31] Shen Z., Denison K., Lobb R., Gatewood J.M., Chen D.J. The human and mouse homologs of the yeast RAD52 gene: cDNA cloning, sequence analysis, assignment to human chromosome 12p12.2-p13, and mRNA expression in mouse tissues. Genomics. 1995; 25:199–206. 7774919 10.1016/0888-7543(95)80126-7

[32] Kagawa W., Kurumizaka H., Ikawa S., Yokoyama S., Shibata T. Homologous pairing promoted by the human Rad52 protein. J. Biol. Chem. 2001; 276:35201–35208. 11454867 10.1074/jbc.M104938200

[33] Lloyd J.A., McGrew D.A., Knight K.L. Identification of residues important for DNA binding in the full-length human Rad52 protein. J. Mol. Biol. 2005; 345:239–249. 15571718 10.1016/j.jmb.2004.10.065

[34] Park M.S., Ludwig D.L., Stigger E., Lee S.H. Physical interaction between human RAD52 and RPA is required for homologous recombination in mammalian cells. J. Biol. Chem. 1996; 271:18996–19000. 8702565 10.1074/jbc.271.31.18996

[35] Shen Z., Cloud K.G., Chen D.J., Park M.S. Specific interactions between the human RAD51 and RAD52 proteins. J. Biol. Chem. 1996; 271:148–152. 8550550 10.1074/jbc.271.1.148

[36] Kagawa W., Kurumizaka H., Ishitani R., Fukai S., Nureki O., Shibata T., Yokoyama S. Crystal structure of the homologous-pairing domain from the human Rad52 recombinase in the undecameric form. Mol. Cell. 2002; 10:359–371. 12191481 10.1016/s1097-2765(02)00587-7

[37] Kagawa W., Kagawa A., Saito K., Ikawa S., Shibata T., Kurumizaka H., Yokoyama S. Identification of a second DNA binding site in the human Rad52 protein. J. Biol. Chem. 2008; 283:24264–24273. 18593704 10.1074/jbc.M802204200 PMC3259773

[38] Grimme J.M., Honda M., Wright R., Okuno Y., Rothenberg E., Mazin A.V., Ha T., Spies M. Human Rad52 binds and wraps single-stranded DNA and mediates annealing via two hRad52-ssDNA complexes. Nucleic Acids Res. 2010; 38:2917–2930. 20081207 10.1093/nar/gkp1249 PMC2875008

[39] Liu J., Doty T., Gibson B., Heyer W.D. Human BRCA2 protein promotes RAD51 filament formation on RPA-covered single-stranded DNA. Nat. Struct. Mol. Biol. 2010; 17:1260–1262. 20729859 10.1038/nsmb.1904 PMC2952495

[40] Jeyasekharan A.D., Liu Y., Hattori H., Pisupati V., Jonsdottir A.B., Rajendra E., Lee M., Sundaramoorthy E., Schlachter S., Kaminski C.F. et al. A cancer-associated BRCA2 mutation reveals masked nuclear export signals controlling localization. Nat. Struct. Mol. Biol. 2013; 20:1191–1198. 24013206 10.1038/nsmb.2666 PMC3796201

[41] Zhao W., Vaithiyalingam S., San Filippo J., Maranon D.G., Jimenez-Sainz J., Fontenay G.V., Kwon Y., Leung S.G., Lu L., Jensen R.B. et al. Promotion of BRCA2-dependent homologous recombination by DSS1 via RPA targeting and DNA mimicry. Mol. Cell. 2015; 59:176–187. 26145171 10.1016/j.molcel.2015.05.032 PMC4506714

[42] Van Komen S., Macris M., Sehorn M.G., Sung P. Purification and assays of Saccharomyces cerevisiae homologous recombination proteins. Methods Enzymol. 2006; 408:445–463. 16793386 10.1016/S0076-6879(06)08028-1

[43] Rothenberg E., Grimme J.M., Spies M., Ha T. Human Rad52-mediated homology search and annealing occurs by continuous interactions between overlapping nucleoprotein complexes. Proc. Natl. Acad. Sci. U.S.A. 2008; 105:20274–20279. 19074292 10.1073/pnas.0810317106 PMC2629295

[44] Ma C.J., Kwon Y., Sung P., Greene E.C. Human RAD52 interactions with replication protein A and the RAD51 presynaptic complex. J. Biol. Chem. 2017; 292:11702–11713. 28551686 10.1074/jbc.M117.794545 PMC5512066

[45] Yamazaki T., Lee W., Arrowsmith C.H., Muhandiram D.R., Kay L.E. A suite of triple-resonance NMR experiments for the backbone assignment of 15N, 13C, 2H-labeled proteins with high sensitivity. J. Am. Chem. Soc. 1994; 116:11655–11666.

[46] Clore G.M., Gronenborn A.M. Multidimensional heteronuclear nuclear magnetic resonance of proteins. Methods Enzymol. 1994; 239:349–363. 7830590 10.1016/s0076-6879(94)39013-4

[47] Yu L., Zhu C.X., Tse-Dinh Y.C., Fesik S.W. Backbone dynamics of the C-terminal domain of Escherichia coli topoisomerase I in the absence and presence of single-stranded DNA. Biochemistry. 1996; 35:9661–9666. 8703937 10.1021/bi960507f

[48] Delaglio F., Grzesiek S., Vuister G.W., Zhu G., Pfeifer J., Bax A. NMRPipe: a multidimensional spectral processing system based on UNIX pipes. J. Biomol. NMR. 1995; 6:277–293. 8520220 10.1007/BF00197809

[49] Johnson B.A., Blevins R.A. NMR View: a computer program for the visualization and analysis of NMR data. J. Biomol. NMR. 1994; 4:603–614. 22911360 10.1007/BF00404272

[50] Grimme J.M., Spies M. FRET-based assays to monitor DNA binding and annealing by Rad52 recombination mediator protein. Methods Mol. Biol. 2011; 745:463–483. 21660711 10.1007/978-1-61779-129-1_27

[51] Stark J.M., Pierce A.J., Oh J., Pastink A., Jasin M. Genetic steps of mammalian homologous repair with distinct mutagenic consequences. Mol. Cell Biol. 2004; 24:9305–9316. 15485900 10.1128/MCB.24.21.9305-9316.2004 PMC522275

[52] Costantino L., Sotiriou S.K., Rantala J.K., Magin S., Mladenov E., Helleday T., Haber J.E., Iliakis G., Kallioniemi O.P., Halazonetis T.D. Break-induced replication repair of damaged forks induces genomic duplications in human cells. Science. 2014; 343:88–91. 24310611 10.1126/science.1243211 PMC4047655

[53] Kamentsky L., Jones T.R., Fraser A., Bray M.A., Logan D.J., Madden K.L., Ljosa V., Rueden C., Eliceiri K.W., Carpenter A.E. Improved structure, function and compatibility for CellProfiler: modular high-throughput image analysis software. Bioinformatics. 2011; 27:1179–1180. 21349861 10.1093/bioinformatics/btr095 PMC3072555

[54] Li J., Zou C., Bai Y., Wazer D.E., Band V., Gao Q. DSS1 is required for the stability of BRCA2. Oncogene. 2006; 25:1186–1194. 16205630 10.1038/sj.onc.1209153

[55] Ochs F., Somyajit K., Altmeyer M., Rask M.B., Lukas J., Lukas C. 53BP1 fosters fidelity of homology-directed DNA repair. Nat. Struct. Mol. Biol. 2016; 23:714–721. 27348077 10.1038/nsmb.3251

[56] Jäntti J., Lahdenranta J., Olkkonen V.M., Söderlund H., Keränen S. SEM1, a homologue of the split hand/split foot malformation candidate gene Dss1, regulates exocytosis and pseudohyphal differentiation in yeast. Proc. Natl. Acad. Sci. U.S.A. 1999; 96:909–914. 9927667 10.1073/pnas.96.3.909 PMC15324

[57] Taylor M.R.G., Špírek M., Chaurasiya K.R., Ward J.D., Carzaniga R., Yu X., Egelman E.H., Collinson L.M., Rueda D., Krejci L. et al. Rad51 paralogs remodel pre-synaptic Rad51 filaments to stimulate homologous recombination. Cell. 2015; 162:271–286. 26186187 10.1016/j.cell.2015.06.015 PMC4518479

[58] Honda M., Okuno Y., Yoo J., Ha T., Spies M. Tyrosine phosphorylation enhances RAD52-mediated annealing by modulating its DNA binding. EMBO J. 2011; 30:3368–3382. 21804533 10.1038/emboj.2011.238 PMC3160658

[59] Huang F., Mazina O.M., Zentner I.J., Cocklin S., Mazin A.V. Inhibition of homologous recombination in human cells by targeting RAD51 recombinase. J. Med. Chem. 2012; 55:3011–3020. 22380680 10.1021/jm201173g

[60] Sullivan K., Cramer-Morales K., McElroy D.L., Ostrov D.A., Haas K., Childers W., Hromas R., Skorski T. Identification of a Small Molecule Inhibitor of RAD52 by Structure-Based Selection. PLoS One. 2016; 11:e0147230. 26784987 10.1371/journal.pone.0147230 PMC4718542

[61] Kristensen C.N., Bystol K.M., Li B., Serrano L., Brenneman M.A. Depletion of DSS1 protein disables homologous recombinational repair in human cells. Mutat. Res. 2010; 694:60–64. 20817001 10.1016/j.mrfmmm.2010.08.007

[62] Schenstrøm S.M., Rebula C.A., Tatham M.H., Hendus-Altenburger R., Jourdain I., Hay R.T., Kragelund B.B., Hartmann-Petersen R. Expanded interactome of the intrinsically disordered protein dss1. Cell Rep. 2018; 25:862–870. 30355493 10.1016/j.celrep.2018.09.080 PMC6218214

[63] Kragelund B.B., Schenstrøm S.M., Rebula C.A., Panse V.G., Hartmann-Petersen R. DSS1/Sem1, a multifunctional and intrinsically disordered protein. Trends Biochem. Sci. 2016; 41:446–459. 26944332 10.1016/j.tibs.2016.02.004

[64] Symington L.S., Gautier J. Double-strand break end resection and repair pathway choice. Annu. Rev. Genet. 2011; 45:247–271. 21910633 10.1146/annurev-genet-110410-132435

[65] Deng S.K., Gibb B., de Almeida M.J., Greene E.C., Symington L.S. RPA antagonizes microhomology-mediated repair of DNA double-strand breaks. Nat. Struct. Mol. Biol. 2014; 21:405–412. 24608368 10.1038/nsmb.2786 PMC3980576

[66] Brouwer I., Zhang H., Candelli A., Normanno D., Peterman E.J.G., Wuite G.J.L., Modesti M. Human RAD52 captures and holds DNA strands, increases DNA flexibility, and prevents melting of duplex DNA: implications for DNA recombination. Cell Rep. 2017; 18:2845–2853. 28329678 10.1016/j.celrep.2017.02.068 PMC5379009

[67] Nik-Zainal S., Davies H., Staaf J., Ramakrishna M., Glodzik D., Zou X., Martincorena I., Alexandrov L.B., Martin S., Wedge D.C. et al. Landscape of somatic mutations in 560 breast cancer whole-genome sequences. Nature. 2016; 534:47–54. 27135926 10.1038/nature17676 PMC4910866

[68] Helleday T., Eshtad S., Nik-Zainal S. Mechanisms underlying mutational signatures in human cancers. Nat. Rev. Genet. 2014; 15:585–598. 24981601 10.1038/nrg3729 PMC6044419

[69] Shima N., Munroe R.J., Schimenti J.C. The mouse genomic instability mutation chaos1 is an allele of Polq that exhibits genetic interaction with Atm. Mol. Cell Biol. 2004; 24:10381–10389. 15542845 10.1128/MCB.24.23.10381-10389.2004 PMC529050

[70] Treuner K., Helton R., Barlow C. Loss of Rad52 partially rescues tumorigenesis and T-cell maturation in Atm-deficient mice. Oncogene. 2004; 23:4655–4661. 15122331 10.1038/sj.onc.1207604

[71] Lieberman R., Xiong D., James M., Han Y., Amos C.I., Wang L., You M. Functional characterization of RAD52 as a lung cancer susceptibility gene in the 12p13.33 locus. Mol. Carcinog. 2016; 55:953–963. 26013599 10.1002/mc.22334 PMC4662629

[72] Lieberman R., You M. Corrupting the DNA damage response: a critical role for Rad52 in tumor cell survival. Aging (Albany NY). 2017; 9:1647–1659. 28722656 10.18632/aging.101263 PMC5559167

[73] Shammas M., Kumar S., Pal J., Nanjappa P., Samur M., Gkotzamanidou M., Shi J., Munshi N.C. Dysregulation of SHFM1, a novel target for prevention of genomic instability in myeloma, is associated with epigenetic changes at specific CpG Sites. Blood. 2014; 124:862.

[74] Hastings P.J., Ira G., Lupski J.R. A microhomology-mediated break-induced replication model for the origin of human copy number variation. PLoS Genet. 2009; 5:e1000327. 19180184 10.1371/journal.pgen.1000327 PMC2621351

[75] Galanos P., Pappas G., Polyzos A., Kotsinas A., Svolaki I., Giakoumakis N.N., Glytsou C., Pateras I.S., Swain U., Souliotis V.L. et al. Mutational signatures reveal the role of RAD52 in p53-independent p21-driven genomic instability. Genome Biol. 2018; 19:37. 29548335 10.1186/s13059-018-1401-9 PMC5857109

