# Supplementary material for: DSS1 interacts with and stimulates RAD52 to promote the repair of DSBs
Source: Nucleic Acids Res. 2019 Dec 4;48(2):694–708. doi: 10.1093/nar/gkz1052 (PMC6954417; doi:10.1093/nar/gkz1052)
Supplement: gkz1052_Supplemental_File [file gkz1052_supplemental_file.docx]

Supplementary

**DSS1 interacts with and stimulates RAD52 to promote the repair of DSBs**

Barbora Stefanovie^1,2,‡^, Sarah R. Hengel^3,‡^, Jarmila Mlcouskova^1,2^, Jana Prochazkova^2^, Mario Spirek^1,2^, Fedor Nikulenkov^2^, Daniel Nemecek^4^, Brandon G. Koch^3^, Fletcher E. Bain^3^, Liping Yu^3,5^, Maria Spies^3^ and Lumir Krejci^1,2,6,*^

^1^Department of Biology, Masaryk University, 62500 Brno, Czech Republic.

^2^International Clinical Research Center, St. Anne’s University Hospital in Brno, 62500 Brno, Czech Republic.

^3^Department of Biochemistry, University of Iowa Carver College of Medicine, 51 Newton Road, Iowa City, Iowa 52242, USA.

^4^CEITEC, Masaryk University, 62500 Brno, Czech Republic

^5^NMR Core Facility, Carver College of Medicine, University of Iowa, Iowa City, Iowa 52242, USA

^6^National Centre for Biomolecular Research, Masaryk University, 62500 Brno, Czech Republic.

Supplementary Figure 1

**Figure S1.** Cell cycle profiles after siRNA treatment and the effect of DSS1 on RAD52 foci formation. **A**. Efficiency of individual siRNA depletions in U2OS cell lines were measured by real-time PCR and normalized to the siCON3 samples. The mean values ± SD from two independent experiments were plotted. **B.** Cell cycle phase distribution following siRNA treatment. U2OS cells were treated with siRNAs and subsequently labeled with PI. Shown is the cell cycle phase percentages for each siRNA treatment, each relative to single cells percentage. **C.** Representative micrographs of YFP-RAD52 foci in U2OS cell nuclei at 2h after exposure to 10 Gy of X-rays. Blue = DAPI, yellow = YFP-RAD52. **D.** Representative micrographs of YFP-RAD52 foci in U2OS cell nuclei at 4h after treatment with 2 mM HU. Blue = DAPI, yellow = YFP-RAD52.

Supplementary Figure 2

**Figure S2.** Characterization of the RAD52-DSS1 interaction. **A.** Full ^15^N/^1^H HSQC spectrum with assigned Trp indole NεH and Asn and Gln sidechain NH2 peaks labelled using DSS1 protein sequence numbering. The dotted rectangle indicates the region where peak assignments are shown in Figure 2D. **B.** Predicted secondary structure probability obtained from the assigned backbone chemical shifts of the DSS1 protein (in the absence of RAD52) using TALOS+ program indicates that residues F52-K62 in DSS1 form an α-helix. **C.** Measured ^15^N{^1^H} heteronuclear NOE. **D.** Measured ^15^N T_2_ relaxation time. The dotted line indicates the average ^15^N T_2_ value for the residues 1-48. Relaxation parameters of ^15^N{^1^H} NOE and ^15^N T_2_ are not shown for the prolines and other residues whose cross-peaks were severely overlapped in the ^15^N/^1^H HSQC spectra. **E.** Quantitative pull-down using a constant amount of GST-DSS1 and increasing amount of RAD52 protein (FT = unbound fractions, B = bound fractions). **F.** GST pull-down assay to test for the interspecies interaction of RAD52 and yeast and human DSS1 homologs. The input (INP) and the bound (B) fractions were analyzed.

Supplementary Figure 3

**Figure S3.** Negative stain electron microscopy of RAD52 and its complexes with DSS1 and/or DNA. Representative top and side views of different RAD52 complexes and the parameters of RAD52 ring estimated from 2D classification in RELION, the scale bar represents 140 nm**.**

Supplementary Figure 4

**Figure S4.** Stopped flow analysis of DNA binding by RAD52 and RAD52-DSS1. **A.** EMSA of fluorescently labelled 30 bp dsDNA incubated with increasing concentration of RAD52 or preformed RAD52-DSS1 complex (1:20). **B.** Protein-dsDNA complexes (with or without crosslinking using 0.1 % glutaraldehyde) are observed as slower migrating bands in agarose gel. Stars indicate position of protein-DNA complexes or free dsDNA. **C.** FRET-based dsDNA binding assay for RAD52 or RAD52-DSS1 (1:20) complex. The dsDNA-RAD52 interaction changes the FRET between Cy3 and Cy5 dyes. Low FRET is observed in the absence of protein; the wrapped state yields high FRET signal; at high RAD52 concentrations, dsDNA can be shared between two or more RAD52 rings yielding lower FRET. Each data point represents average and standard deviation for three independent experiments. Continues line represent fitting of the data to two-step binding. **D.** Fluorescence signal as a function of time for different concentrations of RAD52 binding to Cy3-labelled dT33 ssDNA measured by stopped flow. **E.** Fluorescence signal as a function of time for different concentrations of RAD52-DSS1 binding on Cy3-labelled dT33 ssDNA measured by stopped flow. **F.** Evaluation of stopped flow traces as a change of fluorescence in time. **G.** The dsDNA competition assay monitored by stopped flow. **H.** Evaluation of stopped flow dsDNA competition signals as a change of fluorescence of Cy3-labelled dT33 ssDNA in time. The mean values ± SD from more than three independent experiments were plotted, P < 0.01 = **; ns = not significant.

Supplementary Figure 5

**Figure S5.** D-loop assay with the RAD52, RAD52-DSS1 and RAD51 proteins as a control to compare the D-loop products.

Supplementary Table 1. List of plasmids used in this study.

| **Name** | **Insert** | **Vector backbone** | **Resistance** | **Source** |
| --- | --- | --- | --- | --- |
| pLK271 | GST-DSS1 | pGEX-KG | amp | L. Krejci |
| pLK408 | RAD52 | pET11a | amp | L. Krejci |
| pLK561 | GST-Sem1 | pGEX-6P-1 | amp | L.Krejci |
| pLK1905 | none | pBluescript SK- | amp | L.Krejci |
| ΦX174 | none | ΦX174 RF I | none | New England BioLabs |
| pLK2359 | none | pRSET-B | amp | L.Krejci |
| pLK1711 | I-SceI | pCAGGS | amp | K.Burdova |

Supplementary Table 2. List of DNA substrates used in this study.

| **Name** | **Label** | **Length (nts)** | **Sequence 5’- 3’** | **Application** | **Source** |
| --- | --- | --- | --- | --- | --- |
| pR1027 | 5’-FITC | 33 | ATAGTGTGCAGACTTGGACCCTGCCCTTTCTTT | EMSA,  S1 nuclease protection | VBC Biotech |
| pR101 | 5’-FITC | 61 | GACGCTGCCGAATTCTACCAGTGCCTTGCTAGGACATCTTTGCCCACCTGCAGGTTCACCC | 3’-overhang substrate 1 | VBC Biotech |
| pR2863 | none | 29 | GCAAGGCACTGGTAGAATTCGGCAGCGTC | 3’-overhang substrate 1 | Sigma-Aldrich |
| pR2864 | 3’-Cy5 | 29 | CCTTGGATCTCCGGATAAACTACATAGCC | 3’-overhang substrate 2 | Sigma-Aldrich |
| pR2865 | none | 61 | GGCTATGTAGTTTATCCGGAGATCCAAGGGGGTGAACCTGCAGGTGGGCAAAGATGTCCTA | 3’-overhang substrate 2 | Sigma-Aldrich |
| pR231 | 5’-FITC | 90 | AAATCAATCTAAAGTATATATGAGTAAACTTGGTCTGACAGTTACCAATGCTTAATCAGTGAGGCACCTATCTCAGCGATCTGTCTATTT | D-loop | VBC Biotech |
| T-28-Cy3 | Cy3-internal | 28 | ATAGTTATGGTGAGGACCC/iCy3/CTTTGTTTC | FRET-SSA | IDT DNA Technologies |
| P-28-Cy5 | Cy5-internal | 28 | GAAACAAAGGGGTCC/iCy5/TCACCATAACTAT | FRET-SSA | IDT DNA Technologies |
| dT30 | 5’-Cy3  3’-Cy5 | 30 | TTTTTTTTTTTTTTTTTTTTTTTTTTTTTT | FRET-dsDNA competition assay | IDT DNA Technologies |
| Cy5-28bp-Cy3 | 5’-Cy5  3’-Cy3 | 28 | ATAGTTATGGTGAGGACCCCTTTGTTTC | FRET-dsDNA EMSA | IDT DNA Technologies |
| 28CP | none | 28 | GAAACAAAGGGGTCCTCACCATAACTAT | FRET-dsDNA EMSA | IDT DNA Technologies |
| pR28 | none | 49 | AGCTATGACCATGATTACGAATTGCTTAATTCGTGCAGGCATGGTAGCT | EM | VBC Biotech |

Supplementary Table 3. Summary of siRNA (Ambion) used for gene knock-down.

| **Target gene** | **Name** | **Concentration used** |
| --- | --- | --- |
| DSS1 | **s15512** | 20 nM |
| BRCA2 | **s2085** | 10 nM |
| RAD52 | **s11746** | 40 nM |
| siCON3 | **s444246** | 20 nM |

Supplementary Table 4. List of mammalian cell lines used in this study.

| **Name** | **Purpose** | **Source** |
| --- | --- | --- |
| U2OS-DR | GFP-reporter assay for monitoring HR | J.Stark |
| U2OS-SA | GFP-reporter assay for monitoring SSA | J.Stark |
| U2OS-BIR | GFP-reporter assay for monitoring BIR | T.D. Halazonetis |
| U2OS-YFP-RAD52 | YFP-RAD52 for foci formation assay | C.Lukas |

Supplementary Table 5. Comparison of the kinetic parameters of RAD52 or RAD52-DSS1 mediated annealing in the absence or presence of RPA.

| **[RAD52],**  **nM** | **[RPA],**  **nM** | **[DSS1],**  **nM** | ***k_f_*_ast_ (s^-1^)**  **+/-Error *k_f_*_ast_** | **A_fast_**  **(FRET)** | ***k*_slow_(s^-1^)**  **+/- Error *k*_slow_** | **A_slow_**  **(FRET)** | **ΔFRET** | **%_fast_** | **%_slow_** |
| --- | --- | --- | --- | --- | --- | --- | --- | --- | --- |
| 8 | 0 | 0 | 0.25  +/-0.01 | 0.35 | 0.03  +/-0.001 | 0.31 | 0.66 | 53 | 47 |
| 8 | **2** | 0 | 0.15  +/-0.02 | 0.18 | 0.02  +/-0.003 | 0.47 | 0.38 | 46 | 54 |
| 8 | 0 | **40** | 0.81  +/-0.05 | 0.44 | 0.07  +/-0.004 | 0.21 | 0.65 | 68 | 32 |
| 8 | **2** | **40** | 0.28  +/-0.02 | 0.21 | 0.02  +/-0.001 | 0.46 | 0.34 | 62 | 38 |

Supplementary Table 6. Comparison of the calculated initial rate of annealing (bp/s) of RAD52 or RAD52-DSS1 in the absence or presence of RPA.

| **Protein** |  |  |  |  |
| --- | --- | --- | --- | --- |
| RAD52 | 8 nM | 8 nM | 8 nM | 8 nM |
| RPA | - | 2 nM | - | 2 nM |
| DSS1 | - | - | 40 nM | 40nM |
| **Initial rate (bp/s)** | **5.2** | **5.7** | **17.5** | **12** |

Supplementary Methods:

**Gene silencing by siRNA and Real-Time PCR (RT-PCR)**

Transfections of siRNA duplexes were performed with Lipofectamine RNAiMAX (Thermo Fisher Scientific) at a final concentration of 25 nM for 22h. The efficiency and specificity of siRNAs were tested (**Figure S1A**). For DSS1, RAD52 and BRCA2 knock-downs, 20, 40, and 10 nM siRNA was used, respectively(**Supplementary Table 3**). For the simultaneous knock-down of two genes, combination of concentrations listed above for specific knock-down was used. Nontargeting siRNA (siCON3) was used as control siRNA in all experiments. Total RNA was isolated from the cultured cells on the third day post transfection using High Pure RNA Isolation kit (Roche). Total RNA (280 µg) was reverse transcribed with the Transcriptor First Strand cDNA Synthesis kit (Roche). The expression of mRNAs of specific genes was determined using real-time PCR. Each cDNA sample was amplified using FastStart SYBR Green Master (Roche) on the LightCycler® 480 instrument (Roche). Each reaction contained 8 µL of cDNA and 0.5 µM primers in a final volume of 20 µL. Annealing temperature for BRCA2 mRNA was increased to 61°C compared to the remaining mRNAs, where annealing occurred at 60°C. GAPDH gene was used as an endogenous control to normalize each sample. Relative mRNA expression was further normalized to the control (siCON3). Reported values are averages of two independent experiments.

**Purification of GST-Sem1**

GST-Sem1 was overexpressed in *E.coli* BL21 (DE3) cells. Cells were grown at 37°C until OD_600_=0.6 and then the GST-Sem1 expression was induced by addition of 0.5 mM IPTG and incubation at 37°C for 4h. Harvested cells were resuspended in T+300 buffer, followed by sonication and centrifuged for 1h at 35 000 × g at 4°C. Conductivity of the supernatant was adjusted according to the conductivity of T+100 buffer by dilution using T buffer with no KCl added. Supernatant was loaded on 20-mL Q Sepharose column (GE Healthcare) equilibrated in T+100 buffer. GST-Sem1 was eluted from the column using 10 column volumes of 1-100% KCl gradient of buffer T+1000. Fractions containing GST-Sem1 protein were pooled and mixed with 1mL of Glutathione Sepharose 4B beads equilibrated in T+100 buffer for 1h at 4°C. A gravity column was used to remove unbound fraction and beads were washed with 10 mL of T+100 buffer. GST-Sem1 protein was eluted by 2× 1 mL of T+300 buffer containing 10, 50, 100 or 200 mM glutathione, respectively. The maximum of the protein was eluted by 10 and 50 mM glutathione. All fractions containing GST-Sem1 were pooled, diluted with T buffer with no salt to decrease the conductivity and loaded onto 1-mL Mono Q equilibrated in buffer T+100. GST-Sem1 was eluted with 10 column volume of the 0-100% KCl gradient of T+1000 buffer. Fractions from 300-350 mM KCl were pooled, concentrated on VivaSpin 2,0 (MWCO 5,000) to 150 µL and washed two times in T+100 buffer. Aliquots of concentrated GST-Sem1 were stored at -80°C.

**Purification of GST-DSS1 used in NMR studies**

The GST-DSS1 protein used in NMR studies was expressed in *E. coli*BL21 (DE3) cells in minimal media containing ^15^NH_4_Cl (1g/L) or ^13^C_6_-glucose (0.5g/L) and ^15^NH_4_Cl (1g/L). Cells were grown at 37°C to OD_600_=0.8 for 4 hours and induced with 0.2 mM IPTG. Harvested cells were resuspended in DSS1 Lysis Buffer (100 mM Tris-HCl pH 7.5, 20% sucrose, 4 mM EDTA, 300 mM KCl, 0.01% NP40, 1 mM DTT). Lysis was aided by sonication at amplitude 40 and cell debris was spun down by centrifugation at 10,000 RPM for 1.5 hours at 4°C. Supernatant was filtered through 0.22 µM filter and loaded onto 5-mL GST-trap FF column in GST Buffer (100 mM Tris-HCl, 10% glycerol, 1 mM EDTA, 300 mM KCl, 0.01% NP40, 1 mM DTT). Thrombin (50 units) was added to 5 mL of GST Buffer which was then loaded onto the column to initiate cleavage of GST tag. Cleavage was allowed to proceed overnight at 4°C. DSS1 was eluted by a step elution using GST buffer. Elution was loaded onto a 5-mL Q FF Hi-Trap column equilibrated in Low Salt Buffer (25 mM Tris-HCl, 10% glycerol, 1 mM EDTA, 100 mM KCl, 0.01% NP40, 1 mM DTT). Protein separation was initiated by a 50 mL linear gradient of 100-1000 mM KCl. Peak fractions were concentrated on VivaSpin 6,0 (MWCO 5,000) spin column by centrifuging at 4000 × g for 50 min at 4°C. Protein was aliquoted, flash frozen with liquid nitrogen, and stored at -80°C.

## **Electron microscopy**

RAD52-DSS1 complexes were prepared by preincubation of proteins at stoichiometric ratio at 4°C for 15 min. For ssDNA complexes, RAD52 or preformed RAD52-DSS1 complex were incubated with ssDNA (pR28, **Supplementary Table 2**) in 1:1 ratio for 10 min at 37°C. Protein-DNA mixture was further crosslinked with 0.1% glutaraldehyde for 10 min at RT. Samples of RAD52, RAD52-DSS1 and their ssDNA complexes were diluted in EM buffer (20 mM KH_2_PO_4_ pH 7.5, 150 mM KCl, 0.5 mM EDTA) to ~15 µg/mL protein concentration and 5 µL aliquots applied onto a glow-discharged Quantifoil grid overlaid with thin (~10 nm) continuous carbon. The grids were then washed in deionized H_2_O and negatively stained with nanoW (NanoprobesInc, Yaphank, NY). The specimens were imaged at 50,000× magnification (calibrated) with a FEI Tecnai F20 microscope (FEI, Eindhoven, The Netherlands) at accelerating voltage of 120 kV. Micrographs were recorded on FEI 4k Eagle CCD camera at ~15 e^–^/Å^2^ exposure and 1.0–3.0 μm underfocus.

The contrast transfer function parameters were estimated using program CTFFIND 3(1). Micrographs were manually screened for presence of significant drift and astigmatism and collected particles were 2D classified in RELION 2.1(2).

**Electromobility shift assay (EMSA) with dsDNA**

For dsDNA substrate, the complementary oligonucleotides (Cy5-28bp-Cy3 and 28CP, **Supplementary Table 2**) were annealed by mixing equal parts in the annealing buffer (10 mM Tris pH 7.5, 50 mM NaCl, 1 mM EDTA), heating for 5 min at 95°C and slowly cooling to room temperature. The RAD52 or RAD52-DSS1 complex were added to 10 nM dsDNA at 15, 50, 150, 500, and 1000 nM concentrations in the KPi buffer (30 mM KPi pH 7.4, 1 mM DTT, 0.01 mg/ml BSA). The samples were then split into two and 0.1% glutaraldehyde was added to one half while the same volume of KPi buffer was added to the other half. All samples were incubated at 37°C for 5 minutes. Immediately following incubation, 10x orange loading buffer was added to all samples before loading onto a 1% agarose gel. The agarose gel was run for 1 hour at 50V at room temperature (~22°C). A Bio-Rad ChemiDoc MP Imaging System was used to image the gels in the Cy5 channel. A DSS1 only control, at the same concentrations, in the presence of 10 nM dsDNA was performed to eliminate the possibility of DSS1 alone interacting with the dsDNA.

**Stopped flow ssDNA binding and dsDNA competition**

In the ssDNA binding reactions, two syringes were used for mixing. First syringe contained Cy3 labelled dT33 oligonucleotide (pR1255, **Supplementary table 2**) in a final concentration of 15 nM in 600 µL of Tris-Acetate buffer. Second syringe was gradually filled with increasing concentrations (0.05 – 2.0 µM) of RAD52 or pre-mixed RAD52-DSS1 complex again in 600 µL of Tris-Acetate buffer. RAD52-DSS1 complexes (1:1 ratio) were pre-mixed at room temperature for 7 minutes. The content of both syringes was mixed rapidly, and the fluorescence signal was measured at room temperature. For each concentration, five to seven repetitions were made, averaged and plotted against time.

dsDNA competition using stopped flow technique was performed using Cy3 labelled dT33 oligonucleotide (pR1255, **Supplementary table 2**) at final concentration of 15 nM as described previously. Initially, fluorescence signal was measured for the RAD52 or RAD52-DSS1 pre-mixed complex at concentration of 120 nM (1:8 ratio to ssDNA substrate, calculated from the max FRET value from FRET-based ssDNA binding assay). After stabilization of the signal, 750 nM dsDNA (pRSET B plasmid DNA linearized by PvuII restriction enzyme, **Supplementary Table 1**) was injected to the optical cell and mixed rapidly with RAD52-ssDNA or RAD52-DSS1-ssDNA complexes and the change of fluorescence signal was measured. Again, averages of five to seven independent measurements were plotted as a function of time.

Supplementary references:

1. Mindell, J.A. and Grigorieff, N. (2003) Accurate determination of local defocus and specimen tilt in electron microscopy. *J Struct Biol*, **142**, 334-347.

2. Scheres, S.H. (2012) A Bayesian view on cryo-EM structure determination. *J Mol Biol*, **415**, 406-418.

3. Pettersen, E.F., Goddard, T.D., Huang, C.C., Couch, G.S., Greenblatt, D.M., Meng, E.C. and Ferrin, T.E. (2004) UCSF Chimera--a visualization system for exploratory research and analysis. *J Comput Chem*, **25**, 1605-1612.
